# Serum FFAs profile analysis of Normal weight and obesity individuals of Han and Uygur nationalities in China

**DOI:** 10.1186/s12944-020-1192-3

**Published:** 2020-01-21

**Authors:** Yinghua Ma, Tongtong Qiu, Jiaojiao Zhu, Jingzhou Wang, Xue Li, Yuchun Deng, Xueting Zhang, Jiale Feng, Keru Chen, Cuizhe Wang, Jianxin Xie, Jun Zhang

**Affiliations:** Medical School of Shihezi University, North Second Road, Hongshan Street, Shihezi, 832000 China

**Keywords:** Obesity, Dyslipidemia, FFAs, Metabolic diseases

## Abstract

**Background:**

Han and Uygur are the two main nationalities living in Xinjiang, China. There are significant differences in the incidence of metabolic diseases for two nationalities, but the specific reasons are not clear. Obesity is an important risk factor for the development of metabolic syndrome, which may be closely related to the increase of serum free fatty acids (FFAs) content. This study aims to use metabolomics to compare the changes of serum FFAs profiles between normal weight (NW) and obese (OB) individuals of two nationalities, screening out the differential FFAs, predicting and evaluating their relationship with diseases.

**Methods:**

Thirty-four kinds of FFAs in serum were detected by ultra-high-pressure liquid chromatography–mass spectrometry (UHPLC-MS) and distinctions in FFAs profiles were evaluated using a metabolomics method while Receiver operating characteristics (ROC) and logistic regression models were used to explore FFAs significant for diagnosing obesity and obesity-associated comorbidities.

**Results:**

In the Han nationality, ten kinds of FFAs (C7:0, C8:0, C9:0, C10:0, C11:0, C14:0, C18:2, C20:3, C20:4 and C22:6) showed significant differences between NW and OB individuals. These differential FFAs may be related to hypertension and gestational diabetes mellitus. In the Uygur nationality, C20:3 and C20:5 showed significant differences between NW and OB individuals. C9:0 and C19:0, which were screened out among the female subjects, showed a good ability to predict obesity status in Uygur females (AUC = 0.950).

**Conclusion:**

In both the Han and Uygur nationalities, the FFAs profiles of NW individuals differed from those of OB individuals. The significantly differential FFAs are closely related to obesity and may be important risk factors for obesity and related metabolic diseases.

## Background/introduction

Obesity is associated with several common diseases, such as type 2 diabetes mellitus (T2DM), cardiovascular disease (CVD), stroke, and several types of cancer [1–3]. The worldwide prevalence of obesity is increasing, with approximately 20% of the global adult population predicted to be obese by 2030 [4]. An increase in the prevalence of obesity-related diseases would significantly decrease the quality of life, as well as increase the cost of health care. The Hans and the Uygurs are the main ethnic groups in Xinjiang, China. Due to differences in their culture, lifestyle, diet, and genetic background, the prevalence of obesity and T2DM differ between the two groups. In a survey done in 2016, the prevalence of obesity was reported to be 19.9% in Urumqi, with the Hans, Kazakhs, and Uygurs having prevalences of 12.12, 21.67, and 29.32%, respectively [5]. In 2005, Yan reported that the prevalences of diabetes in the Uygurs according to body mass index (BMI) group were 5.19% (BMI < 24), 6.14% (BMI, 24–28), and 12.27% (BMI ≥ 28) [6]. In 2017, Wang estimated the prevalence of diabetes to be 14.7% in the Chinese Hans and 12.2% in the Uygurs [7].

Due to the unbalanced release and intake of triglycerides (TG) in the muscle, heart, liver, and other tissues, the level of plasma free fatty acids (FFAs) increases in most obese patients [8]. By inhibiting insulin-stimulated glucose uptake and glycogen synthesis, increased plasma FFAs levels are often accompanied by insulin resistance (IR). A large number of reports in the literature have shown that increased levels of FFAs are important risk factors for obesity-related metabolic diseases [9, 10]. Therefore, normalization of plasma FFAs levels is considered as a new treatment for obesity and metabolic diseases [11]. In recent years, with the advancement of metabolomics research, the analysis of the relationship between FFAs and related diseases from the perspective of different subclasses has become a popular issue in this field. For example, sodium palmitate increases intracellular lipid accumulation [12, 13]. Previous study have shown that linoleic acid might have long-term benefits in preventing the onset of T2DM [14]. Some studies have reported that the levels of FFAs vary among different races and sexes [15–17]. However, it remains unclear whether obese (OB) individuals in the Han and Uygur nationalities show significantly different FFAs profiles from normal weight (NW) individuals.

To better understand the relationship between FFAs and obesity metabolic phenotype in the Hans and Uygurs, the present study used a targeted metabolomics method and the ultra-high-pressure liquid chromatography–mass spectrometry (UHPLC-MS) technique. The levels of 34 FFAs were measured in 80 participants. Changes in circulating FFAs profiles in OB individuals were compared with those in NW individuals in the two ethnic groups. For each subject, 9 saturated fatty acids (SFAs); 8 monounsaturated fatty acids (MUFAs); 8 polyunsaturated fatty acids (PUFAs), including ω-3 polyunsaturated fatty acids (ω-3 PUFAs) and ω-6 polyunsaturated fatty acids (ω-6 PUFAs); and 8 odd-carbon fatty acids (OCFAs) were measured (Additional file 1: Tables S2-S7). This study aims to address two specific questions associated with FFAs and obesity. First, is there a significant difference in FFAs characteristics between NW and OB individuals in different ethnic groups? To answer this question, this cross-sectional study measured the difference in FFAs profiles. Second, is there any specific FFAs profiles that predicts the status of obesity and obesity-related diseases? The receiver operating characteristics (ROC) curve was used to assess the ability of FFAs to predict obesity status.

## Materials and methods

### Study design and population

**Fig. 1:**
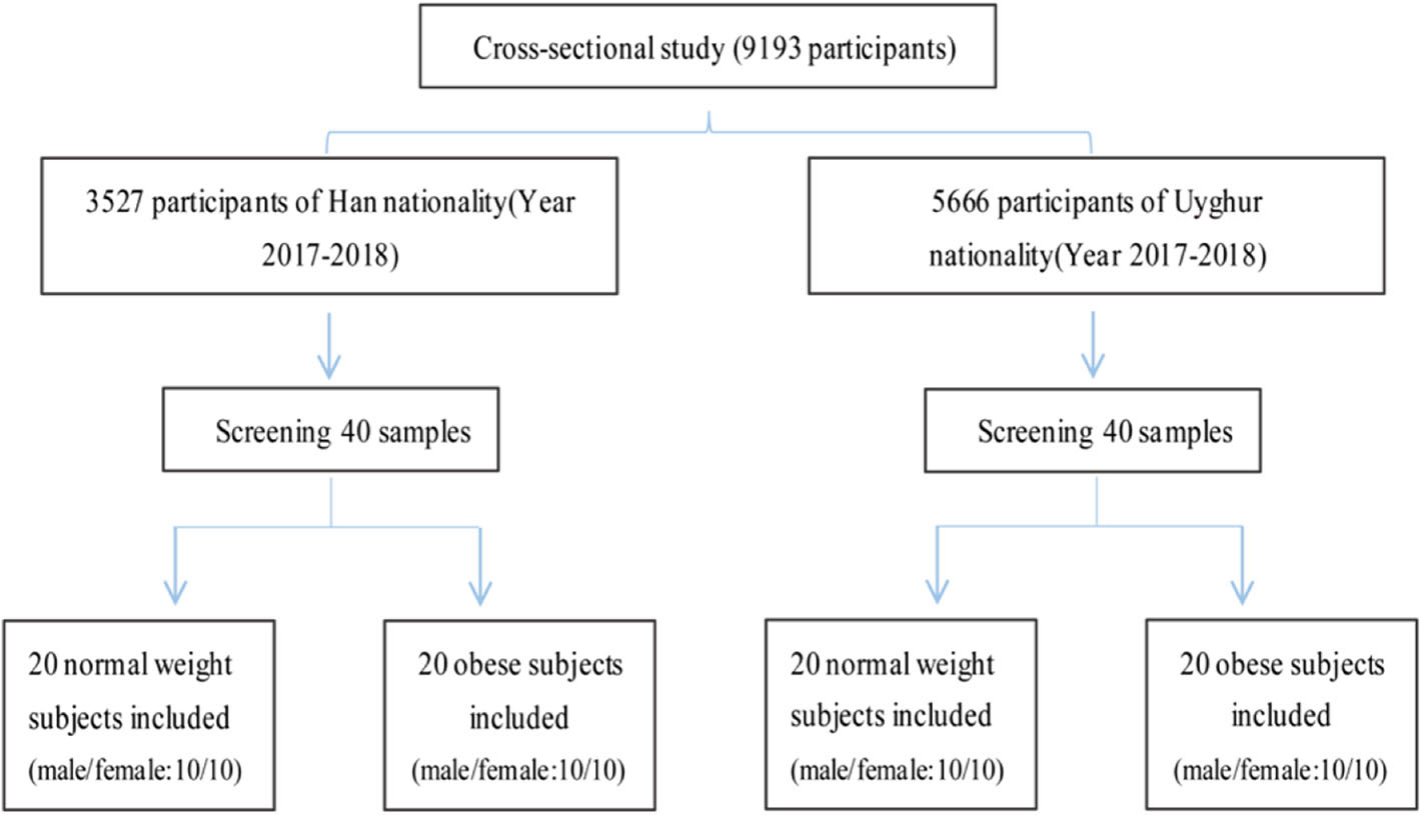
Cross-sectional study used in this analysis

From 2017 to 2018, health examination data were collected from 5666 Uygur adults ages 20 to 65 years in the major Uygur settlements Kashgar, Urumqi, and Turpan, and from 3527 Han adults ages 20 to 65 years in Urumqi and Shihezi (mainly Shihezi city), Xinjiang. From these, 40 samples (*n* = 20 for NW individuals and *n* = 20 for OB individuals) were respective selected from the two ethnic groups according to strict matching conditions on gender, age, and BMI for FFAs analysis. The research flow chart is shown in Fig. 1.

### Human samples and measurement of biochemical indices

General data on height, weight, BMI, waist circumference (WC), systolic blood pressure (SBP), and diastolic blood pressure (DBP) were collected. BMI was calculated according to the formula BMI = body weight (kg)/height (m)^2^. Fasting blood glucose (FPG) was measured by applying the glucose oxidase method. The levels of FPG, total cholesterol (TC), TG, high-density lipoprotein cholesterol (HDL-C), and low-density lipoprotein cholesterol (LDL-C) were determined by using an automatic biochemical analyzer.

Our inclusion criteria are as follows: 40 samples were respective selected from the two ethnic groups for FFAs analysis. The subjects of NW group meet the following conditions: 18.5 ≤ BMI < 24(kg/m^2^), TG ≤ 1.7 mmol / L, TC ≤ 5.2 mmol / L, LDL ≤ 3.4 mmol / L, HDL ≥ 1.0 mmol / L and FPG < 6.1 mmol / L. The subjects of OB group meet the following conditions: BMI ≥ 28(kg/m^2^) and FPG < 6.1 mmol / L.

Subjects with cancer, acute inflammation, liver and kidney disease, and diabetes were not included in the study. The sample we collected also eliminated subjects who had recently taken any medication, including eating medication, doing exercise/ controlling diet to treat the disease.

To avoid circadian rhythm changes, blood samples were taken from 8:00 to 10:30 a.m. after an overnight fast of at least 8 h. The venous blood was centrifuged in a separate gel accelerating tube to separate serum (4000 rpm × 10 min). Part of serum was used to detect the relevant biochemical indicators. The remaining serum was immediately kept at − 80 °C.

### Definition

For the definition of obesity, the present study used the Chinese experts consensus on medical nutrition therapy for overweight/obesity (BMI ≧ 28 kg/m^2^). Before the physical examination, dyslipidemia was diagnosed, or one of its four types, including hypercholesterolemia (TC ≧ 6.2 mmol/L), hypertriglyceridemia (TG ≧ 2.3 mmol/L), hyper-LDL cholesterolemia (LDL-C ≧ 4.1 mmol/L), and hypo-HDL cholesterolemia (HDL-C < 1.0 mmol/L), was diagnosed as dyslipidemia. The criteria recommended by the Guidelines for Prevention and Treatment of Adult Dyslipidemia in China (2016 revised version) were used to determine dyslipidemia.

### UHPLC-MS analysis

FFAs in serum samples were determined by UHPLC-MS on the Tsinghua University metabolomics platform. A 30ul sample was extracted, to which 70ul acetonitrile was added. The solution was then mixed and kept static for 15 min at 4 °C, followed by centrifugation at 12000 rpm for 15–20 min and subsequent removal of the supernatant. Derivatives (20ul) were added to the supernatant to improve the separation degree and detection sensitivity of the mixture. Then, 20ul 1-Ethyl-3-[3-dimethylaminopropyl] carbodiimide was added to improve the coupling efficiency. After mixing, the mixture was briefly centrifuged, followed by incubation at 40 °C for 30 min and centrifugation at 12000 rpm for 15–20 min. The supernatant (20ul), plus 20ul internal standard, was removed, bottled, and analyzed by using an UltiMate 3000 HPLC system.

### Statistical analysis

The data were statistically analyzed by using the SPSS statistical software package version 25.0 (SPSS Inc., Chicago, USA). First, the Shapiro-Wilk normality test was done to evaluate the normality of the data. Then, the Mann-Whitney U test was applied to investigate differences in FFA levels between the NW and OB groups, and 2-way ANOVA was used to analyze the influence of different nationalities and body weight status on biochemical indexes (TC,TG,LDL,HDL,SBP and DBP). Risk factors associated with dyslipidemia, hyperglycemia, and increased blood pressure in the two ethnic groups were analyzed by binary logistic regression. A *p*-value < 0.05 was considered to be statistically significant. All metabolomics analysis plots were done by using the SIMCA 14.1 and the MetaboAnalyst 4.0 software (Umetrics, Sweden). To evaluate the similarities or differences in FFAs curves between the NW and OB groups in the cross-sectional analysis, a supervised multivariate model called the orthogonal partial least squares discriminant analysis (OPLS-DA) was established based on the overall metabolic profile. The ROC curve regions of the FFAs were calculated to assess their ability to distinguish between the NW and OB groups in the cross-sectional study.

## Results

### Subject characteristics

Table 1 presents the clinical profiles of the two ethnic groups. Whether in NW group or OB group, the body weight, BMI and WC of Uygur individuals were signi- ficantly higher than that of Han individuals (*P* < 0.001), and the FPG and TG levels of Uygurs were also significantly higher than those of the Hans (*P* < 0.001). In the NW group, the LDL level of Uygur was significantly higher than that of Hans (*P* < 0.05), while HDL level was significantly lower in the Uygurs than in the Hans (*P* < 0.001). In the OB group, the level of TC and HDL in Uygurs was higher than that in Hans (*P* < 0.05). In addition, we also found that the interaction of the two factors of different nationalities and body weight status could significantly affect the body weight, BMI, FPG and TC of the subjects (*P* < 0.05), and other biochemical indexes (WC, TG and HDL) are affected by two factors (nationality × body weight status) alone, but the interaction of the two factors will not significantly affect these biochemical indexes.

**Table 1:** Comparison of clinical characteristics and biochemical variables between Han and Uygur subjects

Index	Han		Uygur		*P* ^*^
	NW	OB	NW	OB	
Total number of cases	3128	399	3501	2165	
Age (year)	44	47 ^$$$^	42	45 ^$$$##^	0.061
Weight (kg)	60	79 ^$$$^	62 ^###^	80 ^$$$^	0.004
WC (cm)	79	94 ^$$$^	88 ^###^	102 ^$$$###^	0.699
BMI (kg/m^2^)	23.3	29.7 ^$$$^	24.3 ^###^	30.82 ^$$$###^	0.011
FPG (mmol/L)	5.14	5.36 ^$$$^	5.2 ^###^	5.38 ^$$$^	0.046
TG (mmol/L)	1.05	1.49 ^$$$^	1.82 ^###^	2.1 ^$$$###^	0.366
TC (mmol/L)	4.57	4.82 ^$$$^	4.66	4.94 ^$$$#^	0.028
LDL (mmol/L)	2.67	2.83 ^$$^	2.72 ^#^	2.8 ^$$$^	0.636
HDL (mmol/L)	1.44	1.36 ^$$$^	1.32 ^###^	1.38 ^$$$ ##^	< 0.001

Note: The values represent the median .2-way ANOVA test, *P =* significance level of interaction between nationality and body weight status,. * < 0.05; ^$^ significance difference between NW and OB group within the nationality, ^$$^ < 0.01, ^$$$^ < 0.001;

^#^significance difference between Han and Uyghur nationality, ^#^ < 0.05, ^##^ < 0.01, ^# # #^ < 0.001

### Comparison of dyslipidemia detection rates between the two ethnic groups

The detection rates of total dyslipidemia, hypercholesterolemia, hypertriglyceridemia and hypo-HDL cholesterolemia were significantly lower in the Hans than in the Uygurs (*P* < 0.001). These detection rate of dyslipidemias in Han males and females was also significantly lower than that in Uygur males and females (*P* < 0.001). The comparison within ethnic groups showed that these detection rates in Han males was significantly higher than that in females (*P* < 0.001). In contrast, the detection rate in Uygur males was significantly lower than that in females (*P* < 0.001). On the other hand, the detection rate of hyper-LDL cholesterolemia in the Hans was significantly higher than that in the Uygurs (*P* < 0.001). The results of gender analysis showed that the hyper-LDL cholesterolemia detection rate in Han males was also significantly higher than that in Uygur males (*P* < 0.01); no significant differences were found in the comparison of the detection rates of females in the two ethnic groups. The detection rate of hyper-LDL cholesterolemia in Han males was significantly higher than that in females (*P* < 0.001); however, there was no significant difference in detection rate between Uygur males and females. Table 2 shows the results.

**Table 2:** The detection rate of dyslipidemia in Han and Uygur subjects

The Detection Rate	Han			Uygur		
	Male	Female	Total	Male	Female	Total
Hypercholesterolemia (n, %)	5.81 (80/1376)	4.09 (88/2151) ^*^	4.76 (168/3527)	12.18 (278/2283) ^###^	8.90 (301/3383) ^***###^	10.22 (579/5666) ^###^
Hypertriglyceridemia (n, %)	18.10 (249/1376)	7.44 (160/2151) ^***^	11.60 (409/3527	28.56 (652/2283) ^###^	41.35 (1399/3383) ^***###^	36.20 (2051/5666) ^###^
Hyper-LDL cholesterolemia (n, %)	4.07 (56/1376)	1.39 (30/2151) ^***^	2.44 (86/3527)	1.97 (45/2283) ^###^	1.36 (46/3383)	1.61 (91/5666) ^##^
Hypo-HDL cholesterolemia (n, %)	10.83 (149/1376)	4.51(97/2151) ^***^	6.97 (246/3527)	10.78 (246/2283)	20.37 (689/3383) ^***###^	16.50 (935/5666) ^###^
Total dyslipidemia (n, %)	30.01 (413/1376)	15.71 (338/2151) ^***^	21.29 (751/3527)	43.06 (983/2283) ^###^	50.64 (1713/3383) ^***###^	47.58 (2696/5666) ^###^

Note: * These values represent percentages, all *P* values are tested by Chi-square test. *P* < 0.05 is considered statistically significant, *indicates comparison within each ethnic group, *P < 0.05, **P < 0.01, ***P < 0.001; ^#^indicates comparison between 2 ethnic groups, ^###^*P* < 0.001

### Analysis of FFAs profiles of NW and OB individuals in the two nationalities

Thirty-four FFAs were detected by UHPLC-MS, as shown in Additional file 1: Tables S1 to S13. Table 3 describes the demographic characteristics and biochemical indicators of the NW and OB subjects in two nationalities. In the Han nationality, Compared with the NW group, the TG, LDL, SBP, and DBP levels of OB group were significantly higher (*P* < 0.05), whereas the HDL level was significantly lower in OB group. In the Uygur nationality, The TG, SBP, and DBP levels of the OB group were also significantly higher than those of the NW group (*P* < 0.05).

**Table 3:** Demographics

Index	Han(*n* = 40)		Uygur(*n* = 40)	
	NW	OB	NW	OB
Sex (M/F)	10/10	10/10	10/10	10/10
Age (year)	47.15 ± 5.31	47.10 ± 5.16	45.30 ± 1.66	45.05 ± 0.60
Height (cm)	167.50 ± 8.02	167.1 ± 8.10	162.85 ± 5.30	163.35 ± 7.13
Weight (kg)	61.65 ± 5.86	84.20 ± 9.53***	58.35 ± 4.99	83.15 ± 7.43***
BMI (kg/m^2^)	21.95 ± 1.19	30.10 ± 1.53***	21.99 ± 1.3	31.19 ± 2.51***
WC (cm)	83.00 ± 6.09	102.75 ± 4.72***	89.80 ± 6.61	103.45 ± 7.49***
TC (mmol/L)	4.36 ± 0.74	4.64 ± 0.73	4.71 ± 1.30	5.40 ± 1.07
TG (mmol/L)	1.21 ± 0.37	1.85 ± 0.63***	2.09 ± 2.28	2.46 ± 1.43*
LDL (mmol/L)	2.26 ± 0.60	2.73 ± 0.61*	2.44 ± 0.86	2.68 ± 0.92
HDL (mmol/L)	1.33 ± 0.29	1.15 ± 0.14*	1.97 ± 0.65	1.80 ± 0.63
FPG (mmol/L)	5.07 ± 071	5.59 ± 1.86	4.98 ± 0.65	5.15 ± 1.15
SBP (mmHg)	117.95 ± 11.35	136.15 ± 26.33*	119.90 ± 16.48	148.65 ± 32.53***
DBP (mmHg)	73.20 ± 8.46	86.10 ± 16.63**	70.95 ± 10.83	83.60 ± 15.97**

Note: The values represent Mean ± standard deviation; *M* male, *F* female. Nonparametric rank sum test, compared with NW group, *P < 0.05, **P < 0.01, ***P < 0.001

#### Analysis of FFAs profiles of different groups between two nationalities

To determine whether the levels of serum FFAs differs between 20 Han and 20 Uygur individuals in the NW group are different in our metabolomics approach, we constructed two-component OPLS-DA model. The Han and Uygur individuals are obviously divided into two different clusters in the OPLS-DA scores plot independent (Additional file 1: Figure S1a). To ensure that the calculated models are reliable and the observed clustering is not due to chance, we performed an internal validation using 7-fold cross-validation. The calculated goodness of fit (R^2^Y) was 0.976 and the goodness of prediction (Q^2^Y) of 0.923, which underlines the robustness of the model. In addition, a response permutation test to confirm the significance of the prediction ability (Additional file 1: Figure S1b). The results showed that 4 kinds of FFAs increased significantly and 9 kinds of FFAs decreased significantly in the Uygur individuals(*P* < 0.05). The Multivariate analysis showed a variable importance in the projection (VIP) > 1 for 5 kinds of FFAs. Applying VIP > 1 combined with *P* < 0.05 (Table 4), the results confirmed that 2 kinds of FFAs were significantly decreased and one kind of FFA was significantly increased in the serum of Uygur subjects in NW group.

**Table 4:** FFAs profiles analysis of significant difference between Han and Uygur nationality in the NW group

category	Free fatty acid	Normal Weight (NW)		*VIP*^*a*^	*P* Value^b^	*FC*^*c*^
		Han (20)	Uyghur (20)			
MUFAs	C16:1	12.79 ± 11.92	3.68 ± 3.45	**1.31**	**0.008**	**−3.47**
ω-3 PUFAs	C20:5	0.35 ± 0.20	0.18 ± 0.11	**1.67**	**< 0.001**	**−2.00**
OCFAs	C15:0	0.03 ± 0.04	0.40 ± 0.29	**1.25**	**0.032**	**11.46**

*Abbreviations*: *MUFA* monounsaturated fatty acid, *ω-3 PUFA* ω-3 polyunsaturated fatty acid, *OCFA* odd-chain fatty acid, *VIP* Variable importance in the projection, *FC* fold change

^a^The variable importance in the projection (VIP) was obtained in the OPLS-DA. The values in boldface indicate VIP > 1

^b^The *P*-values were calculated from the nonparametric Mann-Whitney U test. The values in boldface indicate P < 0.05

^c^The fold changes (FCs) were calculated from the intra-group means of the FFA levels, with a positive value indicating a relatively higher concentration in the OB group and a negative value indicating a relatively lower concentration compared with the NW group. The numbers in boldface indicate that the absolute FC value is > 1.5

Our results showed that compared with the NW group of Han nationality, the C16:1 (MUFAs) and C20:5 (ω-3 PUFAs) of the NW group of Uygur nationality were significantly decreased, with a geometric mean fold change (FC) of − 3.47 and − 2.0, respectively, and C15:0 (OCFAs) were significantly increased in the NW group of Uyghurs (Table 4, Additional file 1: Figure S1a-S1b). The results of OB group between the two nationalities show that C14:0(SFAs), C16:1 (MUFAs) and C20:5 (ω-3 PUFAs) were significantly decreased in the OB group of Uygurs, with a geometric mean FC of − 1.37,-2.41 and − 1.97, respectively (Table 5, Additional file 1: Figure S1c-S1d).

**Table 5:** FFAs profiles analysis of significant difference between Han and Uygur nationality in the OB group

category	Free fatty acid	Obese (OB)		*VIP*^*a*^	*P* Value^b^	*FC*^*c*^
		Han (20)	Uyghur (20)			
SFAs	C14:0	6.21 ± 2.88	4.54 ± 3.24	**1.51**	**0.023**	−1.37
MUFAs	C16:1	14.88 ± 7.39	6.18 ± 5.17	**1.12**	**0.007**	**−2.41**
ω-3 PUFAs	C20:5	0.61 ± 0.47	0.31 ± 0.22	**1.73**	**0.001**	**−1.97**

*Abbreviations*: *SFA* saturated fatty acid, *MUFA* monounsaturated fatty acid, *ω-6 PUFA* ω-3 PUFA, ω-3 polyunsaturated fatty acid, *VIP* Variable importance in the projection, *FC* fold change

^a^The variable importance in the projection (VIP) was obtained in the OPLS-DA. The values in boldface indicate VIP > 1

^b^The *P*-values were calculated from the nonparametric Mann-Whitney U test. The values in boldface indicate P < 0.05

^c^The fold changes (FCs) were calculated from the intra-group means of the FFA levels, with a positive value indicating a relatively higher concentration in the OB group and a negative value indicating a relatively lower concentration compared with the NW group. The numbers in boldface indicate that the absolute FC value is > 1.5

In the following analysis, we integrated the data of the two ethnic groups, with the purpose of screening out FFAs with significant differences between NW group and OB group among all subjects on the basis of not distinguishing ethnic groups. According to our analysis results, 10 kinds of FFAs in the OB group were significantly increased. Three kinds of ω-6 PUFAs were significantly increased (C18:2, FC = 1.39; C20:3, FC = 1.51 and C20:4, FC = 1.32). Two kinds of ω-3 PUFAs were significantly increased in the OB group compared with the NW group (C18:3, FC = 1.32 and C20:5, FC =1.39). One kind of OCFAs were significantly increased (C11:0, FC = 3.31)(Additional file 1: Table S3, Additional file 1: Figure S1e-S1f).

#### Analysis of FFAs profiles of NW and OB individuals in the Han nationality

To determine whether the levels of serum FFAs differs between 20 NW and 20 OB individuals in our metabolomics approach, we used the same method to analyze the FFAs profiles of NW and OB groups. The results showed that 10 kinds of FFAs were significantly increased in the OB group (*P* < 0.05). The multivariate analysis showed a VIP > 1 for 11 kinds of FFAs. Applying VIP > 1 combined with *P* < 0.05 (Additional file 1: Table S4), the results confirmed that 10 kinds of FFAs were significantly increased in the OB group. Three kinds of SFAs were significantly increased (C8:0, FC = 1.68; C10:0, FC = 1.74; and C14:0, FC = 1.50). Four kinds of ω-6 PUFAs were significantly increased in the OB group compared with the NW group (C18:2, FC = 1.45; C20:3, FC = 1.64; C20:4, FC = 1.39; and C22:6, FC =1.39). Three kinds of OCFAs were significantly increased (C7:0, FC = 3.00; C9:0, FC = 3.29; and C11:0, FC = 3.41). In addition, one kind of ω-3 PUFAs (C22:6) showed a significant increase, with a geometric mean FC of 1.39 (Table 6, Additional file 1: Figure S2a-S2b).

**Table 6:** FFAs profiles analysis of significant difference between the NW and OB groups in two nationalities

Nationality		Category	Free fatty acid	Mean ± standard deviation		VIP^a^	*P* Value^b^	FC^c^
				NW	Obese			
Han	Overall	SFAs	C8:0	0.54 ± 0.27	0.91 ± 0.63	**1.22**	**0.015**	**1.68**
			C10:0	0.21 ± 0.16	0.37 ± 0.28	**1.12**	**0.013**	**1.74**
			C14:0	4.14 ± 2.97	6.21 ± 2.88	**1.14**	**0.013**	**1.50**
		ω-6 PUFAs	C18:2	77.53 ± 35.58	112.10 ± 50.99	**1.25**	**0.025**	1.45
			C20:3	0.86 ± 0.45	1.41 ± 0.95	**1.17**	**0.011**	**1.64**
			C20:4	2.53 ± 1.35	3.51 ± 1.51	**1.11**	**0.033**	1.39
		ω-3 PUFAs	C22:6	2.00 ± 0.86	2.78 ± 1.19	**1.20**	**0.023**	1.39
		OCFAs	C7:0	0.18 ± 0.225	0.55 ± 0.39	**1.66**	**0.001**	**3.00**
			C9:0	2.05 ± 4.16	6.74 ± 5.67	**1.45**	**0.002**	**3.29**
			C11:0	0.06 ± 0.11	0.20 ± 0.16	**1.59**	**0.001**	**3.41**
	Male	SFAs	C12:0	0.37 ± 0.18	0.64 ± 0.27	**1.43**	**0.023**	1.72
			C14:0	3.83 ± 2.23	7.18 ± 2.90	**1.51**	**0.013**	1.88
		ω-6 PUFAs	C18:2	69.45 ± 33.15	125.66 ± 62.21	**1.50**	**0.016**	1.81
			C20:3	0.73 ± 0.23	1.64 ± 1.22	**1.42**	**0.013**	2.25
			C20:4	2.25 ± 1.11	3.99 ± 1.34	**1.59**	**0.008**	1.77
		ω-3PUFAs	C22:6	1.97 ± 0.81	2.98 ± 1.30	**1.21**	**0.049**	**1.51**
	Female	SFAs	C8:0	0.47 ± 0.15	0.75 ± 0.31	**1.30**	**0.049**	**1.61**
			C10:0	0.18 ± 0.09	0.31 ± 0.15	**1.24**	**0.049**	**1.69**
		OCFAs	C7:0	0.18 ± 0.18	0.57 ± 0.30	**1.99**	**0.003**	**3.15**
			C9:0	1.35 ± 1.98	7.26 ± 4.69	**2.05**	**0.006**	**5.37**
			C11:0	0.04 ± 0.07	0.24 ± 0.14	**2.23**	**0.001**	**5.17**
Uygur	Overall	ω-6 PUFAs	C20:3	0.88 ± 0.64	1.22 ± 0.71	**1.24**	**0.023**	1.39
		ω-3 PUFAs	C20:5	0.18 ± 0.11	0.31 ± 0.22	**1.04**	**0.020**	**1.76**
	Female	OCFAs	C9:0	0.07 ± 0.04	0.16 ± 0.06	**1.55**	**0.014**	**1.78**
			C19:0	0.03 ± 0.03	0.09 ± 0.04	**1.97**	**0.009**	**1.94**

Abbreviations: *SFA* saturated fatty acid, *MUFA* monounsaturated fatty acid, *ω-6* PUFA ω-6 polyunsaturated fatty acid, *ω-3* PUFA ω-3 polyunsaturated fatty acid, *OCFA* odd-chain fatty acid, *VIP* Variable importance in the projection, *FC* fold change

^a^The variable importance in the projection (VIP) was obtained in the OPLS-DA. The values in boldface indicate VIP > 1

^b^The P-values were calculated from the nonparametric Mann-Whitney U test. The values in boldface indicate P < 0.05

^c^The fold changes (FCs) were calculated from the intra-group means of the FFA levels, with a positive value indicating a relatively higher concentration in the OB group and a negative value indicating a relatively lower concentration compared with the NW group. The numbers in boldface indicate that the absolute FC value is > 1.5

A similar metabolomics study was done in males and females (Additional file 1: Figure S2c-S2f). Among the males, the results showed that six kinds of FFAs were significantly increased in the OB group. Two kinds of SFAs (C12:0, FC = 1.72 and C14:0, FC = 1.88) and three kinds of ω-6 PUFAs (C18:2, FC = 1.81; C20:3, FC = 2.25; and C20:4, FC = 1.77) were significantly increased in the OB group compared with the NW group. In contrast, only one kind of ω-3 PUFAs (C22:6) showed a significant increase, with a geometric mean FC of 1.51(Table 6, Additional file 1: Figure S2c-S2d). Among the females, the results showed that five kinds of FFAs were significantly increased in the OB group. Two kinds of SFAs (C8:0, FC = 1.61 and C10:0, FC = 1.69) and three kinds of OCFAs (C7:0, FC = 3.15; C9:0 FC = 5.37; and C11:0, FC = 5.17) were significantly increased in the OB group compared with the NW (Table 6, Additional file 1: Figure S2e-S2f). The same method was used to compare and analyze the FFAs profiles between different genders in the Han nationality, the results showed that there was no significant difference in FFAs between male NW group and female NW group (Additional file 1: Table S7, Additional file 1: Figure S3a-S3b). In the OB group, the content of C24:0 was significantly lower in females than in males (FC = -1.55) (Table 7, Additional file 1: Figure S3c-S3d).

**Table 7:** FFAs profiles analysis of significant difference between the male and female groups in OB group of Han nationality

category	Free fatty acid	Obese (OB)		*VIP*^*a*^	*P* Value^b^	*FC*^*c*^
		Male (10)	Female (10)			
SFAs	C24:0	0.54 ± 0.17	0.35 ± 0.19	**1.35**	**0.041**	**−1.55**

*Abbreviations*: *SFA* saturated fatty acid, *VIP* Variable importance in the projection, *FC* fold change

^a^The variable importance in the projection (VIP) was obtained in the OPLS-DA. The values in boldface indicate VIP > 1

^b^The *P*-values were calculated from the nonparametric Mann-Whitney U test. The values in boldface indicate *P* < 0.05

^c^The fold changes (FCs) were calculated from the intra-group means of the FFA levels, with a positive value indicating a relatively higher concentration in the OB group and a negative value indicating a relatively lower concentration compared with the NW group. The numbers in boldface indicate that the absolute FC value is > 1.5

#### Analysis of FFAs profiles of NW and OB individuals in the Uygur nationality

The overall results showed that C20:3 (ω-6 PUFAs) and C20:5 (ω-3 PUFAs) were significantly increased in the OB group, with a geometric mean FC of 1.39 and 1.76, respectively (Table 6, Additional file 1: Figure S4a-S4b). Among the males, there was no significant difference in FFAs between the NW and OB groups (Additional file 1: Figure S4c-S4d; Additional file 1: Table S10). Among the females, the results indicated that two of the nine kinds of OCFAs were significantly increased in the OB group compared with the NW group (C9:0, FC = 1.78 and C19:0, FC = 1.94) (Table 6, Additional file 1: Figure S4e-S4f).

The same method was used to compare and analyze the FFAs profiles between different genders in the Uygur nationality, in the NW group, our results showed significantly decreased in two kinds of SFAs(C8:0, FC = -1.57 and C10:0, FC = -2.06) and one kind of OCFAs were significantly increased (C9:0, FC = -2.39) in females than in males (Table 8, Additional file 1: Figure S5a-S5b). In the OB group, there was no significant difference in FFAs between the males and females groups (Additional file 1: Table S13, Additional file 1: Figure S5c-S5d).

**Table 8:** FFAs profiles analysis of significant difference between the male and female groups in NW group of Uygur nationality

category	Free fatty acid	Normal Weight (NW)		*VIP*^*a*^	*P* Value^b^	*FC*^*c*^
		Male (10)	Female (10)			
SFAs	C8:0	0.81 ± 0.36	0.52 ± 0.23	**1.09**	**0.041**	**−1.57**
	C10:0	0.40 ± 0.23	0.19 ± 0.13	**1.72**	**0.034**	**−2.06**
OCFAs	C9:0	0.16 ± 0.08	0.07 ± 0.04	**1.58**	**0.013**	**−2.39**

*Abbreviations*: *SFA* saturated fatty acid, *OCFA* odd-chain fatty acid, *VIP* Variable importance in the projection, *FC* fold change

^a^The variable importance in the projection (VIP) was obtained in the OPLS-DA. The values in boldface indicate VIP > 1

^b^The *P*-values were calculated from the nonparametric Mann-Whitney U test. The values in boldface indicate *P* < 0.05

^c^The fold changes (FCs) were calculated from the intra-group means of the FFA levels, with a positive value indicating a relatively higher concentration in the OB group and a negative value indicating a relatively lower concentration compared with the NW group. The numbers in boldface indicate that the absolute FC value is > 1.5

In order to explore whether these FFAs with significant differences are common or unique to the two nationalities, the further analysis result showed that only C20:3 was common to the two nationalities, the remaining eight kinds of FFAs are unique to the Han nationality, and one kind of FFA is unique to the Uygur nationality (Additional file 1: Figure S6a). Among the females, only C9:0 was common to the two nationalities. The four other kinds of FFAs were unique to the Hans, whereas C19:0 was unique to the Uygurs (Additional file 1: Figure S6b).

### Metabolomics assessment as a diagnostic test for obesity in the two nationalities

#### Metabolomics assessment as a diagnostic test for obesity in the Han nationality

**Fig. 2:**
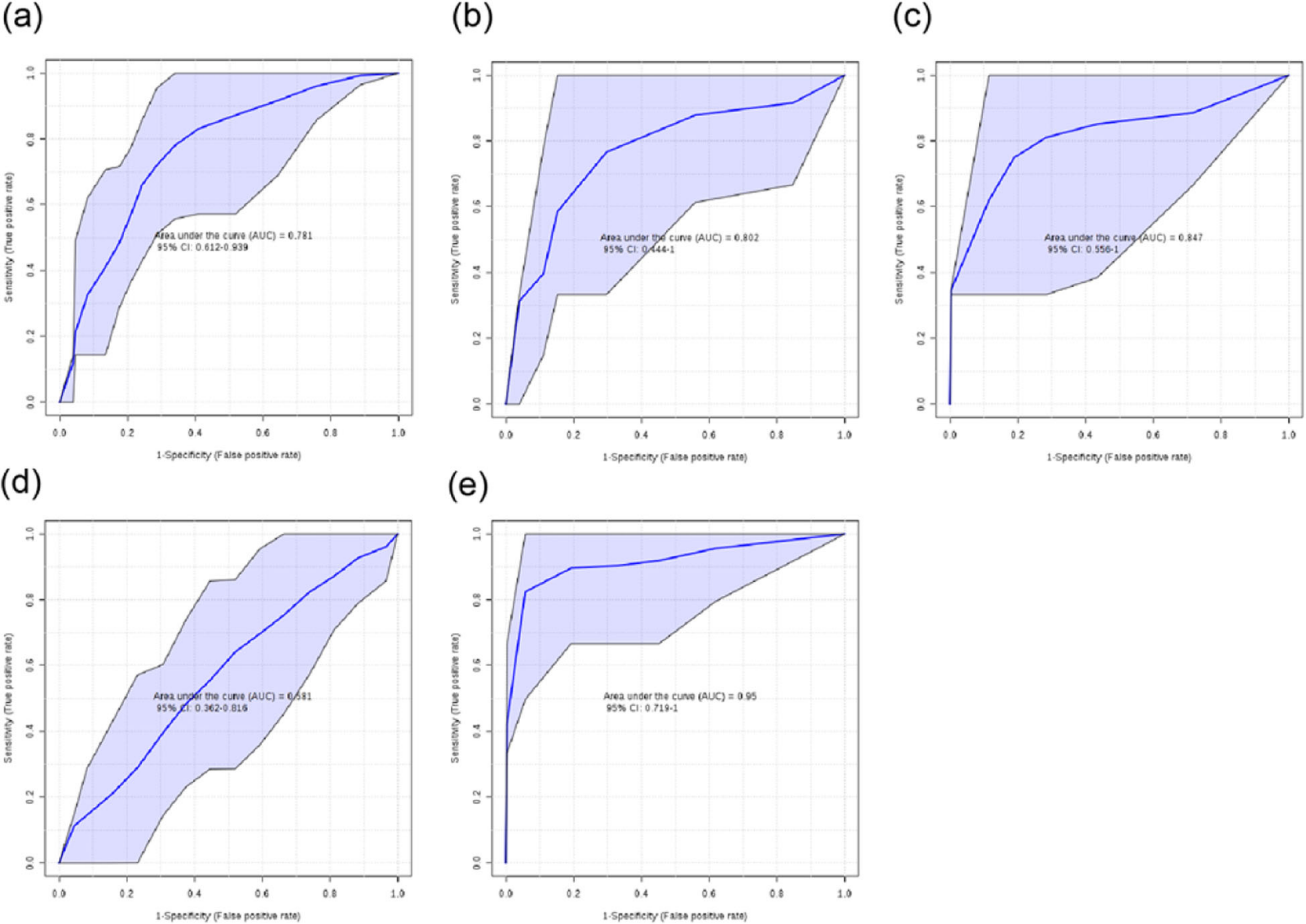
The diagnostic performance of targeted metabolomics in obesity in two nationalities; AUROC curve analysis. **a**11 FFAS with significant differences in OB group were used to predict obesity status in Han nationality,the 11 FFAs were C7:0,C8:0,C9:0,C10:0, C11:0, C14:0, C18:2,C20:3,C20:4 and C22:6.The diagnostic accuracy measured as the AUROC curve was 0.781 (95% CI,0.612–0.939). **b** Males, 6 FFAS with significant differences in OB group were used to predict obesity status in Han males, the 6 FFAs were C12:0, C14:0, C18:2, C20:3,C20:4 and C22:6.The diagnostic accuracy measured as the AUROC curve was 0.847 (95% CI, 0.556–1.0). **c** Females, 5 FFAS with significant differences in OB group were used to predict obesity status in Han females, the 5 FFAs were C7:0,C8:0,C9:0,C10:0 and C11:0.The diagnostic accuracy measured as the AUROC curve was 0.847 (95% CI, 0.556–1.0), **d** A significant increase in C20:3 and C20:5 in OB group was used to predict obesity status in Uygur nationality. The diagnostic accuracy measured as the AUROC curve was 0.581 (95% CI, 0.362–0.816), **e** Females, A significant increase in C9:0 and C19:0 in OB group was used to predict obesity in Uygur females. The diagnostic accuracy measured as the AUROC curve was 0.950 (95% CI, 0.719–1.0)

Based on the above results, several FFAs that showed significant differences between the NW and OB groups were screened out and defined as smaller analyte groups for the diagnosis of the status of obesity. The performance of each classifier metabolite set was then tested by analyzing the area under the receiver operating characteristic (AUROC) curve. Overall, ten kinds of FFAs that showed differences in the OB group were defined as the analysis group, capable of diagnosing obesity in the Han nationality (AUC = 0.781; Fig. 2a). Among the males, six analytes showed a good performance (AUC = 0.802; Fig. 2b). Among the females, a group of five analytes also showed a good performance (AUC = 0.847; Fig. 2c). The results indicated that even the single analyte classification method showed a certain performance in this small sample of 40 subjects (Additional file 1: Table S14).

#### Metabolomics assessment as a diagnostic test for obesity in the Uygur nationality

Overall, two FFAs showed significant differences between the NW and OB groups. However, these two FFAs did not achieve good results when they were used to diagnose the overall obesity status of the Uygur subjects (AUC = 0.581; Fig. 2d). Among the females, the analyte group was defined by two different FFAs with very good performances (AUC = 0.950; Fig. 2e). The results showed a good performance by even a single analyte classification method in a small sample of 20 women (Additional file 1: Table S14).

### Multivariate linear regression analysis between the two nationalities

Multiple linear regression analysis was used to analyze the relation between the FFAs levels and the risk factors for related biochemical indicators (TC, TG, LDL, HDL, FPG, SBP, and DBP).

#### Multivariate linear regression analysis in the Han nationality

The results showed that even excluding the effects of age, BMI, and WC, one or more FFAs were closely related to the biochemical indicators. Among them, C20:3 and C11:0 were independently correlated with changes in TC, C20:3 was a positive risk factor for TC increase (β = 0.297, 95%CI =0.003–0.591), and C11:0 was a negative risk factor for TC increase (β = − 1.775, 95%CI = (− 3.345) - (− 0.204)). In addition, C18:2 was independently correlated with changes in LDL and was a positive risk factor for LDL increase (β = 0.005, 95%CI =0.001–0.009). Table 9 shows the results.

**Table 9:** Multivariate linear regression analysis between the two nationalities

		Dependent variable	Β	t	*P*	Exp(B)95%CI	S. E	Partial correlation
Han	TC	constant	4.162	20.909	<0.001		0.199	
		C20:3	0.297	2.047	0.048	0.003–0.591	0.145	0.315
		C11:0	−1.775	−2.290	0.028	(−3.345)-(−0.204)	0.775	−0.169
	LDL	constant	2.007	9.213	<0.001	1.566–2.448	0.218	
		C18:2	0.005	2.482	0.018	0.001–0.009	0.002	0.373
Uygur	TC	constant	4.162	20.909	<0.001	3.795–4.565	0.199	
		C20:3	6.297	2.047	0.048	0.003–0.591	0.145	0.315
	LDL	constant	2.148	12.689	0.000	1.805–2.490	0.169	
		C20:3	0.305	2.468	0.018	0.055–0.554	0.123	0.372
	FPG	constant	4.920	16.848	0.000	4.329–5.512	0.292	
		C19:0	11.194	2.061	0.046	0.200–22.187	5.431	0.371

Note: The multivariate linear regression analysis excluded the effects of age, BMI and waist circumference on the model

#### Multivariate linear regression analysis in the Uygur nationality

Similarly, in the Uygur nationality, C20:3 was independently correlated with TC changes and was a positive risk factor for TC increase (β = 6.297, 95%CI =0.003–0.591). In addition, C20:3 was a positive risk factor for LDL increase (β = 0.305, 95%CI =0.055–0.554). C19:0 was a positive risk factor for FPG and showed a high correlation with FPG increase (β = 11.194, 95%CI =0.200–22.187). Table 9 shows the results.

### Pathway analysis of obesity-related FFAs and analysis of enriched diseases in the two nationalities

#### Pathway analysis of obesity-related FFAs and analysis of enriched diseases in the Han nationality

**Fig. 3:**
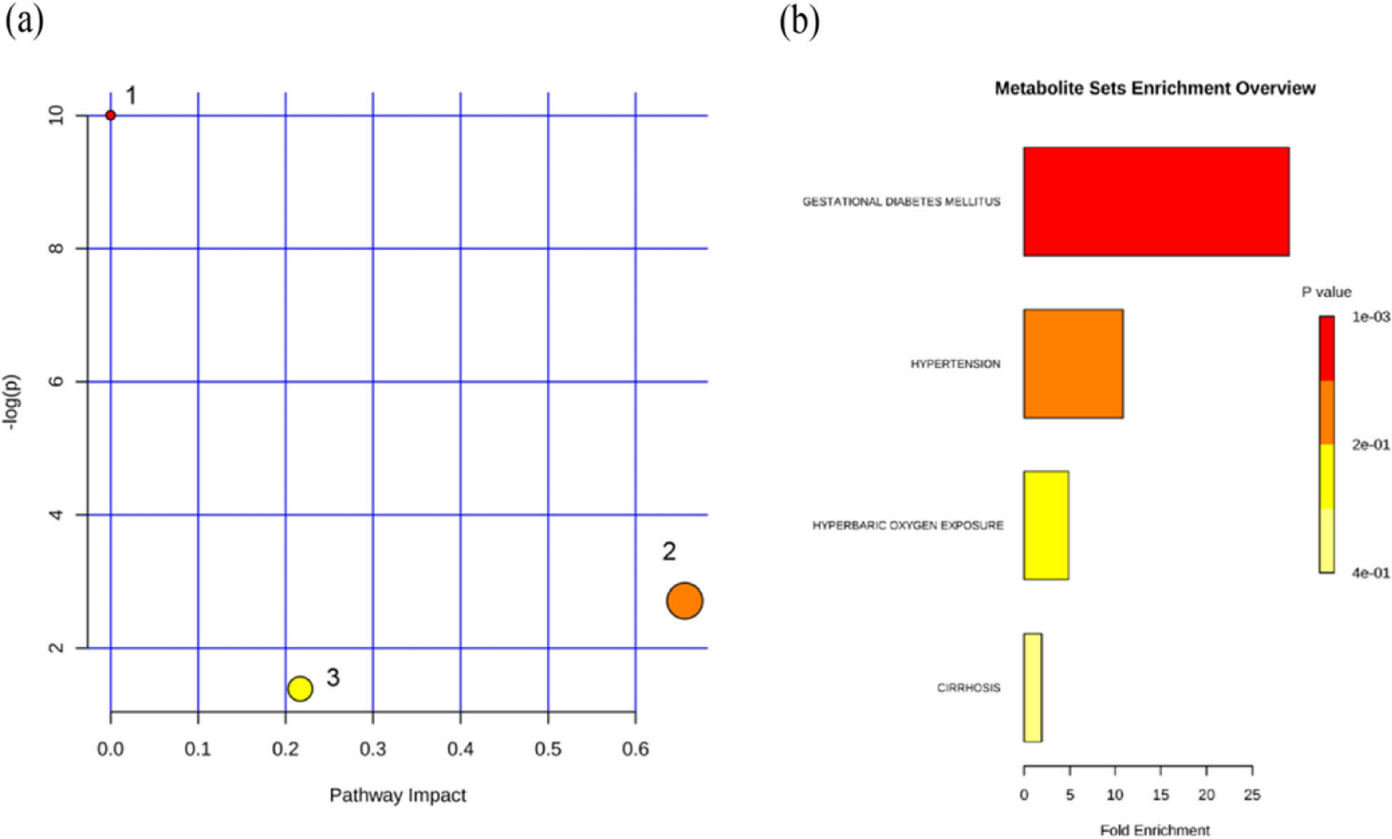
Relevant pathways and diseases involved in FFAs with significant differences between NW and OB groups. **a** Abnormal pathway in differential FFAs group, as Analyzed by MetaboAnalyst, (1) Fatty acid biosynthesis pathway, (2) Linoleic acid metabolism, (3) Arachidonic acid metabolism pathway, **b** Analysis of disease enrichment in differential FFAs

Based on the knowledge of the differential FFAs in the Han nationality and by using KEGG pathways to analysis. The result showed that three related pathways were found to be disturbed under obesity. In the present study, the original *p*-value was set to 0.05, and the impact was set to 0.1 for the significant pathway. Three kinds of FFAs (C8:0, C10:0, and C14:0) participated in the fatty acid biosynthesis pathway, which satisfied *p* < 0.05 but not satisfied impact > 0.1. C18:2 participated in the linoleic acid metabolism pathway, and C20:4 participated in the arachidonic acid metabolism pathway. The linoleic acid and arachidonic acid metabolism pathways only satisfied impact > 0.1 (Fig. 3a, Additional file 1: Table S15). The results indicated that several pathways were disrupted in the pathologic process of obesity. Therefore, by applying disease enrichment analysis to elucidate the relationship between distinct FFAs and obesity-related diseases, the initial *P*-value was set to 0.05, and the false discovery rate (FDR) of significant related diseases was set to 0.1. The enrichment analysis results showed that the FFA contents were enriched in four diseases, among which hypertension was consistent with *P* < 0.05 and FDR < 0.1, and gestational diabetes mellitus was consistent only with *P* < 0.05 (Fig. 3b, Additional file 1: Table S16). In addition, changes in FFAs were found to be associated with various obesity-related diseases.

#### Pathway analysis of obesity-related FFAs and analysis of enriched diseases in the Uygur nationality

Previous studies have shown significant differences in some FFAs between NW and OB groups. The pathway analysis indicated that these FFAs with significant differences were not involved in any metabolic pathways. The enrichment analysis showed the same results.

## Discussion

In China, the obesity prevalence between 1980 and 2015 increased in both males and females, reaching 10.53% in 2015 [18]. The present results showed that the detection rates of obesity in the Han and Uygur nationalities in Xinjiang were 11.31 and 38.21%, respectively, which were higher than the national level, especially that in the Uygurs. The results of our study on two main ethnic groups, namely, the Hans and Uygurs, showed dyslipidemia detection rates of 21.29 and 47.58%, respectively. Obesity causes metabolic disorders in the body, which in turn leads to abnormal blood lipid levels [19].

In recent years, an increasing number of studies have focused on the changes in FFAs levels in subjects with different health conditions. Recently, Yan Ni et al. analyzed the FFAs profiles in serum of normal weight (NW) and healthy obese (HO) subjects. The results showed that the levels of SFAs and ω-3 PUFAs in the HO group were higher than those in the NW group, whereas the levels of ω-6 PUFAs were lower than those in the NW group [20].

The present study analyzed for the first time the profiles of 34 kinds of FFAs in NW and OB individuals of Han and Uygur nationalities in Xinjiang, China. The levels of these FFAs in most subjects in the OB group were found to be higher than those in the NW group, and significant differences in some FFAs levels were observed between the NW and OB groups. More concretely, in the Han nationality, the levels of C7:0, C8:0, C9:0, C10:0, C11:0, C12:0, C14:0, C18:2, C20:3, C20:4 and C22:6 in the OB group were significantly higher than those in the NW group. In the Uygurs, the levels of C20:3 and C20:5 in the OB group were significantly higher than those in the NW group. In the Uygur females, the levels of C9:0 and C19:0 in the OB group were significantly higher than those in the NW group. These findings suggest that obesity could increase the serum levels of various FFAs.

A large number of works in the literature have reported a relationship between some kinds of FFAs and certain diseases. For instance, the C20:5 and C22:6 may counteract the adipokine dysregulation that occurs in obesity and related diseases, such as T2DM [21]. Furthermore, Jenkins et al. showed that saturated even-numbered fatty acids have a strong positive association with T2DM incidence and increased risk of coronary heart disease (CHD), whereas plasma OCFAs showed a strong inverse association with disease risk and CHD incidence [22]. Our results suggest that C20:4 and C22:6 are associated with gestational diabetes mellitus, meanwhile, C18:3, C20:4 and C22:6 are associated with hypertension. The FFAs that showed significant differences between the NW and OB groups in the present study are closely related to obesity-related diseases, thus providing a new direction and research basis for follow-up studies.

BMI has been widely used as a reliable and simple index for the measurement of obesity. However, many individuals with normal BMI are described as having “metabolic obesity but normal weight” (MONW) [23]. Suliga et al. reported that 17.27% of normal weight patients were diagnosed with metabolic syndrome [24]. In China in particular, despite having a low absolute BMI, individuals are more likely to accumulate visceral fat and IR compared with those in the West. According to the relevant literature, there are limited data on the prevention of T2DM among the normal weight population in China. The triglyceride glucose (TyG) index has been used as an indicator of risk of incident IR and T2DM [25]. Therefore, TG may be a potential indicator for predicting metabolic disorders in vivo, and the circulating FFA content is closely related to the uptake and release of TG.

The present study used, for the first time, a set of FFAs to predict the obesity status in a small sample of Han and Uygur individuals in Xinjiang. The results indicated that even a single analyte classification method has the ability to predict obesity status. However, single biomarkers are biologically unbelievable as diagnostic tests for obesity-related complex diseases and may perform poorly in larger populations. The use of a classifier consisting of metabolites makes it easier to adapt to natural biological variations and results in more robust diagnostic accuracy. Our results indicated that in the Hans, the different FFAs analysis groups have some accuracy in predicting obesity status. In the Uygurs, the differential FFAs analysis groups have poor predictive ability for obesity status in the whole sample but have good predictive ability among the females. Interestingly, only OCFAs showed significant increases in the Uygur female OB group compared with the NW group. This suggests that OCFAs (C9:0 and C19:0) could be used as an effective marker for predicting obesity and related diseases in Uygur women. Given the small sample, there may be some limitations in predicting obesity in both ethnic groups based on the FFAs analysis group. The need for a larger sample to verify metabolically obese and obesity-associated comorbidities are therefore a key issue in follow-up reasearch.

In addition, it has been reported that diet, exercise habits and nutritional status all affect the blood lipids levels and composition of subjects [26–29]. The focus of present study is to screen out FFAs with significant differences between NW and OB individuals of different nationalities, and these different FFAs may be a potential biomarker for predicting metabolic obesity and related diseases. Therefore, in our present study, we did not pay attention to the impact of diet, exercise habits and nutritional status on the FFAs level of the two ethnic groups, which is worth further investigation in the future research.

## Conclusion

The main advantage of the present work lies in its comprehensive design. For the first time, FFAs that showed significant changes between NW and OB subjects in Xinjiang Hans and Uygurs were screened out, and their involvement in pathways to obesity and related diseases was analyzed. In addition, a set of FFAs was used to predict obesity in both ethnic groups for the first time. This study also provides a new theoretical basis for the prevention of obesity and related diseases. In future works, the limitation presented by the small sample size will be addressed by using a larger sample for further verification of the obtained results.

## Supplementary information

**Additional file 1: Table S1.** Significant differences in FFAs detected by UHPLC-MS and analyzed by OPLS-DA and Mann-Whitney U test between the Han and Uyghur nationalities in NW group. **Table S2.** Significant differences in FFAs detected by UHPLC-MS and analyzed by OPLS-DA and Mann-Whitney U test between the Han and Uyghur nationalities in OB group. **Table S3.** Significant differences in FFAs detected by UHPLC-MS and analyzed by OPLS-DA and Mann-Whitney U test between the NW and OB groups in all individuals of two nationalities. **Table S4.** 34 FFAs detected by UHPLC-MS and analyzed by OPLS-DA and the Mann-Whitney U-test in NW and OB group of Han nationality. **Table S5.** 34 FFAs detected by UHPLC-MS and analyzed by OPLS-DA and the Mann-Whitney U-test in NW and OB group of Han males. **Table S6.** 34 FFAs detected by UHPLC-MS and analyzed by OPLS-DA and the Mann-Whitney U-test in NW and OB group of Han females. **Table S7.** Significant differences in FFAs detected by UHPLC-MS and analyzed by OPLS-DA and Mann-Whitney U test between the male and female groups in NW group of Han nationality. **Table S8.** Significant differences in FFAs detected by UHPLC-MS and analyzed by OPLS-DA and Mann-Whitney U test between the male and female groups in OB group of Han nationality. **Table S9.** 34 FFAs detected by UHPLC-MS and analyzed by OPLS-DA and the Mann-Whitney U-test in NW and OB group of Uygur. **Table S10.** 34 FFAs detected by UHPLC-MS and analyzed by OPLS-DA and the Mann-Whitney U-test in NW and OB group of Uygur males. **Table S11.** 34 FFAs detected by UHPLC-MS and analyzed by OPLS-DA and the Mann-Whitney U-test in NW and OB group of Uygur females. **Table S12.** Significant differences in FFAs detected by UHPLC-MS and analyzed by OPLS-DA and Mann-Whitney U test between the male and female groups in NW group of Uyghur nationality. **Table S13.** Significant differences in FFAs detected by UHPLC-MS and analyzed by OPLS-DA and Mann-Whitney U test between the male and female groups in the OB group of Uygur nationality. **Figure S1.** Orthogonal partial least squares discriminant analysis in different groups between two nationalities. (a)The score plot of the OPLS-DA model shows a clear discrimination between the 20 NW subjects of Hans (green triangle) and 20 NW subjects of Uygurs (blue square), (b)Permutation test with a permutation number of 200 in the NW groups between two nationalities, (c)The score plot of the OPLS-DA model shows a clear discrimination between the 20 OB subjects of Hans and 20 OB subjects of Uygurs, (d)Permutation test with a permutation number of 200 in the OB groups between two nationalities, (e)The score plot of the OPLS-DA model shows a clear discrimination between the 40 NW subjects and 40 OB subjects of two nationalities, (f)Permutation test with a permutation number of 200 in different groups between two nationalities. **Figure S2.** Orthogonal partial least squares discriminant analysis between NW and OB group in Han nationality. (a)The score plot of the OPLS-DA model shows a clear discrimination between the 20 OB subjects (blue square)(blue square) and 20 NW subjects (green triangle)(green circle) in the Han nationality, (b)Permutation test with a permutation number of 200 in Han nationality, (c)The score plot of the OPLS-DA model shows a clear discrimination between the 10 OB subjects and 10 NW subjects in the male group, (d)Permutation test with a permutation number of 200 in the male group, (e)The score plot of the OPLS-DA model shows a clear discrimination between the 10 OB subjects and 10 NW subjects in the female group, (f)Permutation test with a permutation number of 200 in the female group. **Figure S3.** Orthogonal partial least squares discriminant analysis between different genders in the Han nationality. (a)The score plot of the OPLS-DA model shows a clear discrimination between the 10 males (green triangle) and 10 females (blue square) in the NW group (b)Permutation test with a permutation number of 200 in the NW group, (c)The score plot of the OPLS-DA model shows a clear discrimination between the 10 males and 10 females in the OB group, (d)Permutation test with a permutation number of 200 in the OB group. **Figure S4.** Orthogonal partial least squares discriminant analysis between normal NW OB group in Uygur nationality. (a)The score plot of the OPLS-DA model shows a clear discrimination between the 20 OB subjects (green triangle)(blue square) and 20 NW subjects (blue square)(green circle) in the Uygur nationality, (b)Permutation test with a permutation number of 200 in the Uygur nationality, (c)The score plot of the OPLS-DA model shows a discrimination between the 10 OB subjects and 10 NW subjects in the males group, (d)Permutation test with a permutation number of 200 in the males group, (e)The score plot of the OPLS-DA model shows a clear discrimination between the 10 OB subjects and 10 NW subjects in the females group, (f)Permutation test with a permutation number of 200 in the females group. **Figure S5.** Orthogonal partial least squares discriminant analysis between different genders in the Uygur nationality. (a)The score plot of the OPLS-DA model shows a clear discrimination between the 10 males (green triangle) and 10 females (blue square) in the NW group (b)Permutation test with a permutation number of 200 in the NW group, (c)The score plot of the OPLS-DA model shows a clear discrimination between the 10 males and 10 females in the OB group, (d)Permutation test with a permutation number of 200 in the OB group. **Figure S6.** The traditional Venn diagram is used to observe the generality and personality differences of FFAs between NW and OB group in two nationalities. (a)On the overall level, observing the generality and personality differences of FFAs between NW and OB group in two nationalities, (b)In the male group, observing the generality and personality differences of FFAs between NW and OB group in two nationalities. **Table S14.** Diagnostic accuracy of targeted plasma metabolomics in status of obesity. **Table S15.** Pathway enrichment analysis of FFAs. **Table S16.** Disease enrichment analysis of FFAs.

## Data Availability

Data and all materials supporting this research are available from the corresponding author on reasonable request.

## Abbreviations

AUROC: Area under the receiver operating characteristic
BMI: Body mass index
CHD: Coronary heart disease
CI: Confidence interval
CVD: Cardiovascular disease
DBP: Diastolic blood pressure
FBG: Fasting blood glucose
FC: Fold change
FDR: False discovery rate
FFAs: Free fatty acids
HDL-C: High-density lipoprotein cholesterol
IR: Insulin resistance
LDL-C: Low-density lipoprotein cholesterol
MONW: Metabolic obesity but normal weight
MUFAs: Monounsaturated fatty acids
NW: Normal weight
OB: Obese
OCFAs: Odd-carbon fatty acids
OPLS-DA: Orthogonal partial least squares discriminant analysis
PUFAs: Polyunsaturated fatty acids
ROC: Receiver operating characteristics
SBP: Systolic blood pressure
SFAs: Saturated fatty acids
T2DM: Type 2 diabetes mellitus
TC: Total cholesterol
TG: Triglyceride
TyG: Triglyceride glucose
UHPLC-MS: Ultra-high-pressure liquid chromatography-mass spectrometry
VIP: Variable importance in the projection
WC: Waist circumference
ω-3 PUFAs: ω-3 polyunsaturated fatty acids
ω-6 PUFAs: ω-6polyunsaturated fatty acids
