# Supplementary material for: Serum FFAs profile analysis of Normal weight and obesity individuals of Han and Uygur nationalities in China
Source: Lipids Health Dis. 2020 Jan 21;19:13. doi: 10.1186/s12944-020-1192-3 (PMC6975073; doi:10.1186/s12944-020-1192-3)
Supplement: Supplementary file 1 — Additional file 1: Table S1. Significant differences in FFAs detected by UHPLC-MS and analyzed by OPLS-DA and Mann-Whitney U test between the Han and Uyghur nationalities in NW group. Table S2. Significant differences in FFAs detected by UHPLC-MS and analyzed by OPLS-DA and Mann-Whitney U test between the Han and Uyghur nationalities in OB group. Table S3. Significant differences in FFAs detected by UHPLC-MS and analyzed by OPLS-DA and Mann-Whitney U test between the NW and OB groups in all individuals of two nationalities. Table S4. 34 FFAs detected by UHPLC-MS and analyzed by OPLS-DA and the Mann-Whitney U-test in NW and OB group of Han nationality. Table S5. 34 FFAs detected by UHPLC-MS and analyzed by OPLS-DA and the Mann-Whitney U-test in NW and OB group of Han males. Table S6. 34 FFAs detected by UHPLC-MS and analyzed by OPLS-DA and the Mann-Whitney U-test in NW and OB group of Han females. Table S7. Significant differences in FFAs detected by UHPLC-MS and analyzed by OPLS-DA and Mann-Whitney U test between the male and female groups in NW group of Han nationality. Table S8. Significant differences in FFAs detected by UHPLC-MS and analyzed by OPLS-DA and Mann-Whitney U test between the male and female groups in OB group of Han nationality. Table S9. 34 FFAs detected by UHPLC-MS and analyzed by OPLS-DA and the Mann-Whitney U-test in NW and OB group of Uygur. Table S10. 34 FFAs detected by UHPLC-MS and analyzed by OPLS-DA and the Mann-Whitney U-test in NW and OB group of Uygur males. Table S11. 34 FFAs detected by UHPLC-MS and analyzed by OPLS-DA and the Mann-Whitney U-test in NW and OB group of Uygur females. Table S12. Significant differences in FFAs detected by UHPLC-MS and analyzed by OPLS-DA and Mann-Whitney U test between the male and female groups in NW group of Uyghur nationality. Table S13. Significant differences in FFAs detected by UHPLC-MS and analyzed by OPLS-DA and Mann-Whitney U test between the male and female groups in the OB group of Uygur nati [file 12944_2020_1192_MOESM1_ESM.docx]

**Additional File**

Yinghua Ma ^#^, Tongtong Qiu ^#^, Jiaojiao Zhu , Jingzhou Wang , Xue Li , Yuchun Deng ^,^ Xueting Zhang , Jiale Feng , Keru Chen ^,^ Cuizhe Wang *, Jianxin Xie * and Jun Zhang *

Serum FFAs Profile Analysis of Normal Weight and Obesity Individuals of Han and Uygur Nationalities in China

Medical school of Shihezi University, North Second Road, Hongshan Street, Shihezi 832000,China;[2489187019@qq.com(Y.M.);](mailto:2489187019@qq.com(Y.M.);)[1505925808@qq.com(T.Q.);](mailto:1505925808@qq.com(T.Q.);) [1648977156@qq.com(J.Z.);](mailto:1648977156@qq.com(J.Z.);)344930369@qq.com(X.Z.); 2464127505@qq.com(J.F.); 1090816950@qq.com(K.C.)

* Correspondence: zhangjunyc@163.com (J.Z.); xiejianxin9017@sina.com (J.X.); wangcuizhe905@163.com (C.W.); Tel.: +86-188-0993-7979(J.Z.); +86-135-1993-6016(J.X.); +86-152-9943-1968(C.W.);

# Contributed equally.

**Table S1.** Significant differences in FFAs detected by UHPLC-MS and analyzed by OPLS-DA and Mann-Whitney U test between the Han and Uyghur nationalities in NW group.

| **category** | **Free fatty acid** | **Normal Weight(NW)**  **Han(20) Uyghur(20)** | | ***VIP^a^*** | ***P* Value^b^** | ***FC^c^*** |
| --- | --- | --- | --- | --- | --- | --- |
| **SFAs** | C8:0 | 0.54±0.27 | 0.66±0.33 | **1.13** | 0.261 | 1.23 |
|  | C10:0 | 0.21±0.16 | 0.29±0.21 | 0.51 | **0.020** | 1.38 |
|  | C12:0 | 0.47±0.24 | 0.40±0.40 | 0.80 | **<0.001** | -1.17 |
|  | C14:0 | 4.14±2.97 | 3.49±2.75 | 0.69 | 0.337 | -1.18 |
|  | C16:0 | 716.09±351.34 | 40.54±29.88 | 0.43 | 0.490 | **-17.66** |
|  | C18:0 | 0 | 0 | 0 | 1 | 0 |
|  | C20:0 | 0.21±0.19 | 0.62±1.31 | 0.18 | **0.009** | **2.99** |
|  | C22:0 | 0 | 0 | 0 | 1 | 0 |
|  | C24:0 | 0.48±0.29 | 0.36±0.14 | 0.16 | **0.020** | -1.32 |
| **MUFAs** | C14:1 | 1.70±3.51 | 0 | 0.11 | **<0.001** | 0 |
|  | C15:1 | 0 | 0 | 0 | 1 | 0 |
|  | C16:1 | 12.79±11.92 | 3.68±3.45 | **1.31** | **0.008** | **-3.47** |
|  | C17:1 | 0.12±0.11 | 7.76 | 0.74 | **0.002** | **63.02** |
|  | C18:1 | 106.89±73.79 | 30.37±18.26 | 0.95 | 0.441 | **-3.52** |
|  | C20:1 | 1.57±1.59 | 0.16±0.14 | 0.26 | **0.003** | **-9.59** |
|  | C22:1 | 0.07±0.20 | 0 | 0.14 | 1 | 0 |
|  | C24:1 | 0.09±0.04 | 0.12±0.04 | 0.82 | 0.694 | 1.36 |
| **ω-6 PUFAs** | C18:2 | 77.53±35.58 | 37.58±26.56 | 0.52 | 0.394 | **-2.06** |
|  | C20:2 | 3.07±2.42 | 0.59±0.68 | 0.56 | **0.042** | **-5.15** |
|  | C20:3 | 0.86±0.45 | 0.88±0.64 | 0.01 | 0.285 | 1.02 |
|  | C20:4 | 2.53±1.35 | 1.81±0.82 | 0.19 | 0.330 | -1.40 |
|  | C22:2 | 0 | 0.03±0.03 | 0 | 1 | 0 |
| **ω-3 PUFAs** | C18:3 | 1.39±0.66 | 0.51±0.26 | 0.57 | **0.016** | **-2.74** |
|  | C20:5 | 0.35±0.20 | 0.18±0.11 | **1.67** | **<0.001** | **-2.00** |
|  | C22:6 | 2.00±0.86 | 1.17±0.77 | 0.27 | 0.552 | **-1.70** |
| **OCFAs** | C7:0 | 0.18±0.225 | 0.11±0.07 | **1.14** | 0.222 | -1.62 |
|  | C9:0 | 2.05±4.16 | 0.11±0.08 | 0.32 | 0.292 | **-18.23** |
|  | C11:0 | 0.06±0.11 | 0.01±0.01 | 0.77 | **0.005** | **-4.66** |
|  | C13:0 | 0.05±0.04 | 0.02±0.02 | 0.32 | 0.521 | **-2.01** |
|  | C15:0 | 0.03±0.04 | 0.40±0.29 | **1.25** | **0.032** | **11.46** |
|  | C17:0 | 0.26±0.28 | 1.02±0.68 | 0.76 | 0.490 | **3.89** |
|  | C19:0 | 0.03±0.04 | 0.04±0.05 | 0.85 | 0.369 | 1.28 |
|  | C21:0 | 0 | 0 | 0 | 1 | 0 |
|  | C23:0 | 0 | 0 | 0 | 1 | 0 |

Abbreviations: SFA, saturated fatty acid; MUFA, monounsaturated fatty acid; ω-6 PUFA, ω-6 polyunsaturated fatty acid; ω-3 PUFA, ω-3 polyunsaturated fatty acid; OCFA, odd-chain fatty acid; VIP, Variable importance in the projection; FC, fold change.

a The variable importance in the projection (VIP) was obtained in the OPLS-DA. The values in boldface indicate VIP>1.

b The *P*-values were calculated from the nonparametric Mann-Whitney U test. The values in boldface indicate P<0.05.

c The fold changes (FCs) were calculated from the intra-group means of the FFA levels, with a positive value indicating a relatively higher concentration in the OB group and a negative value indicating a relatively lower concentration compared with the NW group. The numbers in boldface indicate that the absolute FC value is >1.5.

**Table S2.** Significant differences in FFAs detected by UHPLC-MS and analyzed by OPLS-DA and Mann-Whitney U test between the Han and Uyghur nationalities in OB group.

| **category** | **Free fatty acid** | **Obese(OB)**  **Han(20) Uyghur(20)** | | ***VIP^a^*** | ***P* Value^b^** | ***FC^c^*** |
| --- | --- | --- | --- | --- | --- | --- |
| **SFAs** | C8:0 | 0.91±0.63 | 0.69±0.51 | 0.43 | **0.001** | -1.30 |
|  | C10:0 | 0.37±0.28 | 0.30±0.27 | 0.62 | **0.001** | -1.24 |
|  | C12:0 | 0.56±0.26 | 0.48±0.36 | 0.93 | **<0.001** | -1.15 |
|  | C14:0 | 6.21±2.88 | 4.54±3.24 | **1.51** | **0.023** | -1.37 |
|  | C16:0 | 979.19±588.36 | 53.06±35.68 | 0.50 | 0.482 | **-18.45** |
|  | C18:0 | 0 | 0 | 0 | 1 | 0 |
|  | C20:0 | 0.22±0.14 | 0.61±1.02 | 0.06 | **0.001** | **2.79** |
|  | C22:0 | 0 | 0 | 0 | 1 | 0 |
|  | C24:0 | 0.44±0.20 | 0.42±0.26 | 0.56 | 0.093 | -1.05 |
| **MUFAs** | C14:1 | 1.34±2.36 | 0 | 0.17 | **<0.001** | 0 |
|  | C15:1 | 0 | 0 | 0 | 1 | 0 |
|  | C16:1 | 14.88±7.39 | 6.18±5.17 | **1.12** | **0.007** | **-2.41** |
|  | C17:1 | 0.16±0.10 | 0.40±0.38 | 0.60 | **<0.001** | **2.50** |
|  | C18:1 | 147.14±79.61 | 40.11±24.70 | **1.06** | 0.117 | **-3.67** |
|  | C20:1 | 1.67±1.40 | 0.14±0.10 | 0.78 | **<0.001** | **-12.15** |
|  | C22:1 | 0.03±0.10 | 0 | 0.37 | 1 | 0 |
|  | C24:1 | 0.08±0.03 | 0.11±0.06 | 0.67 | 0.953 | 1.37 |
| **ω-6 PUFAs** | C18:2 | 112.10±50.99 | 47.42±27.99 | 0.08 | 0.358 | **-2.36** |
|  | C20:2 | 3.89±2.21 | 0.71±0.65 | 0.24 | **0.006** | **-5.50** |
|  | C20:3 | 1.41±0.95 | 1.22±0.71 | 0.17 | 0.351 | -1.16 |
|  | C20:4 | 3.51±1.51 | 2.24±1.13 | 0.08 | **0.042** | **-1.57** |
|  | C22:2 | 0 | 0.01±0.00 | 0 | 1 | 0 |
| **ω-3 PUFAs** | C18:3 | 1.71±0.71 | 0.81±0.55 | 0.07 | **0.030** | **-2.11** |
|  | C20:5 | 0.61±0.47 | 0.31±0.22 | **1.73** | **0.001** | **-1.97** |
|  | C22:6 | 2.78±1.19 | 1.36±0.83 | 0.99 | 0.213 | **-2.04** |
| **OCFAs** | C7:0 | 0.55±0.39 | 0.11±0.08 | **1.00** | 0.369 | **-5.02** |
|  | C9:0 | 6.74±5.67 | 0.17±0.08 | 0.70 | 0.249 | **-40.12** |
|  | C11:0 | 0.20±0.16 | 0.02±0.01 | 0.68 | 0.820 | **-11.13** |
|  | C13:0 | 0.08±0.06 | 0.03±0.01 | 0.85 | 0.054 | **-2.99** |
|  | C15:0 | 0.05±0.05 | 0.49±0.35 | **1.29** | 0.079 | **9.80** |
|  | C17:0 | 0.36±0.25 | 1.25±0.82 | 0.85 | 0.570 | **3.50** |
|  | C19:0 | 0.04±0.04 | 0.07±0.05 | 0.20 | 0.055 | **1.73** |
|  | C21:0 | 0 | 0 | 0 | 1 | 0 |
|  | C23:0 | 0.09±0.42 | 0 | 0 | 1 | 0 |

Abbreviations: SFA, saturated fatty acid; MUFA, monounsaturated fatty acid; ω-6 PUFA, ω-6 polyunsaturated fatty acid; ω-3 PUFA, ω-3 polyunsaturated fatty acid; OCFA, odd-chain fatty acid; VIP, Variable importance in the projection; FC, fold change.

a The variable importance in the projection (VIP) was obtained in the OPLS-DA. The values in boldface indicate VIP>1.

b The *P*-values were calculated from the nonparametric Mann-Whitney U test. The values in boldface indicate P<0.05.

c The fold changes (FCs) were calculated from the intra-group means of the FFA levels, with a positive value indicating a relatively higher concentration in the OB group and a negative value indicating a relatively lower concentration compared with the NW group. The numbers in boldface indicate that the absolute FC value is >1.5.

**Table S3.** Significant differences in FFAs detected by UHPLC-MS and analyzed by OPLS-DA and Mann-Whitney U test between the NW and OB groups in all individuals of two nationalities.

| **category** | **Free fatty acid** | **Mean ± standard deviation**  **NW(40) OB(40)** | | ***VIP^a^*** | ***P* Value^b^** | ***FC^c^*** |
| --- | --- | --- | --- | --- | --- | --- |
| **SFAs** | C8:0 | 0.60±0.30 | 0.80±0.58 | 0.84 | 0.232 | 1.33 |
|  | C10:0 | 0.25±0.19 | 0.33±0.27 | 0.62 | 0.104 | 1.32 |
|  | C12:0 | 0.44±0.32 | 0.52±0.31 | 0.47 | 0.553 | 1.18 |
|  | C14:0 | 3.83±2.84 | 5.38±3.14 | 0.91 | **0.010** | 1.41 |
|  | C16:0 | 386.98±423.27 | 516.13±623.86 | 0.95 | 0.056 | 1.33 |
|  | C18:0 | 0 | 0 | 0 | 1 | 0 |
|  | C20:0 | 0.34±0.74 | 0.32±0.53 | 0.10 | 0.757 | -1.04 |
|  | C22:0 | 0 | 0 | 0 | 1 | 0 |
|  | C24:0 | 0.42±0.23 | 0.43±0.23 | 0.09 | 0.846 | 1.02 |
| **MUFAs** | C14:1 | 1.70±3.51 | 1.34±2.36 | 0.13 | 0.408 | -1.22 |
|  | C15:1 | 0 | 0 | 0 | 1 | 0 |
|  | C16:1 | 8.23±9.81 | 10.53±7.68 | 0.58 | **0.028** | 1.28 |
|  | C17:1 | 0.49±1.67 | 0.18±0.14 | 0.10 | 0.099 | **-2.65** |
|  | C18:1 | 68.63±65.70 | 93.62±79.51 | 0.92 | 0.028 | 1.36 |
|  | C20:1 | 1.17±1.49 | 1.13±1.34 | 0.05 | 0.192 | -1.04 |
|  | C22:1 | 0.07±0.20 | 0 | 0.29 | 0.215 | **-2.45** |
|  | C24:1 | 0.10±0.05 | 0.09±0.05 | 0.30 | 0.508 | -1.09 |
| **ω-6 PUFAs** | C18:2 | 57.56±37.01 | 79.76±52.17 | **1.10** | **0.012** | 1.39 |
|  | C20:2 | 1.83±2.16 | 2.30±2.29 | 0.54 | 0.090 | 1.26 |
|  | C20:3 | 0.87±0.55 | 1.31±0.83 | **1.21** | **0.001** | **1.51** |
|  | C20:4 | 2.17±1.16 | 2.88±1.47 | **1.08** | **0.014** | 1.32 |
|  | C22:2 | 0.003±0.01 | 0.0005±0.002 | 0.32 | 0.501 | **-6.00** |
| **ω-3 PUFAs** | C18:3 | 0.95±0.67 | 1.26±0.78 | **1.15** | **0.021** | 1.32 |
|  | C20:5 | 0.26±0.18 | 0.46±0.39 | **1.01** | **0.025** | 1.39 |
|  | C22:6 | 1.59±0.91 | 2.07±1.24 | 0.90 | **0.049** | 1.31 |
| **OCFAs** | C7:0 | 0.15±0.19 | 0.40±0.38 | **1.01** | 0.228 | **2.63** |
|  | C9:0 | 1.22±3.26 | 4.55±5.56 | **1.36** | 0.070 | **3.73** |
|  | C11:0 | 0.04±0.09 | 014±0.16 | **1.25** | **0.001** | **3.31** |
|  | C13:0 | 0.04±0.04 | 0.06±0.06 | 0.58 | 0.246 | 1.47 |
|  | C15:0 | 0.21±0.27 | 0.27±0.33 | 0.52 | 0.403 | 1.28 |
|  | C17:0 | 0.64±0.64 | 0.80±0.75 | 0.61 | **0.041** | 1.25 |
|  | C19:0 | 0.04±0.04 | 0.05±0.05 | 0.66 | 0.369 | 1.37 |
|  | C21:0 | 0 | 0 | 0 | 1 | 0 |
|  | C23:0 | 0 | 0.09±0.42 | 0 | 1 | 0 |

Abbreviations: SFA, saturated fatty acid; MUFA, monounsaturated fatty acid; ω-6 PUFA, ω-6 polyunsaturated fatty acid; ω-3 PUFA, ω-3 polyunsaturated fatty acid; OCFA, odd-chain fatty acid; VIP, Variable importance in the projection; FC, fold change.

a The variable importance in the projection (VIP) was obtained in the OPLS-DA. The values in boldface indicate VIP>1.

b The *P*-values were calculated from the nonparametric Mann-Whitney U test. The values in boldface indicate P<0.05.

c The fold changes (FCs) were calculated from the intra-group means of the FFA levels, with a positive value indicating a relatively higher concentration in the OB group and a negative value indicating a relatively lower concentration compared with the NW group. The numbers in boldface indicate that the absolute FC value is >1.5.

**Table S4.** 34 FFAs detected by UHPLC-MS and analyzed by OPLS-DA and the Mann-Whitney U-test in NW and OB group of Han nationality.

| **category** | **Free fatty acid** | **Mean±standard deviation**  **NW (20) OB (20)** | | ***VIP^a^*** | ***P* Value^b^** | ***FC^c^*** |
| --- | --- | --- | --- | --- | --- | --- |
| **SFAs** | C8:0 | 0.54±0.27 | 0.91±0.63 | **1.22** | **0.015** | **1.68** |
|  | C10:0 | 0.21±0.16 | 0.37±0.28 | **1.12** | **0.013** | **1.74** |
|  | C12:0 | 0.47±0.24 | 0.56±0.26 | 0.54 | 0.317 | 1.17 |
|  | C14:0 | 4.14±2.97 | 6.21±2.88 | **1.14** | **0.013** | **1.50** |
|  | C16:0 | 716.09±351.34 | 979.19±588.36 | 0.88 | 0.176 | 1.37 |
|  | C18:0 | 0 | 0 | 0 | 1 | 0 |
|  | C20:0 | 0.21±0.19 | 0.22±0.14 | 0.11 | 0.507 | 1.06 |
|  | C22:0 | 0 | 0 | 0 | 1 | 0 |
|  | C24:0 | 0.48±0.29 | 0.44±0.20 | 0.29 | 0.914 | -0.13 |
| **MUFAs** | C14:1 | 1.70±3.51 | 1.34±2.36 | 0.20 | 0.828 | -0.34 |
|  | C15:1 | 0 | 0 | 0 | 1 | 0 |
|  | C16:1 | 12.79±11.92 | 14.88±7.39 | 0.36 | 0.148 | 1.16 |
|  | C17:1 | 0.12±0.11 | 0.16±0.10 | 0.62 | 0.074 | 1.31 |
|  | C18:1 | 106.89±73.79 | 147.14±79.61 | 0.87 | 0.083 | 1.38 |
|  | C20:1 | 1.57±1.59 | 1.67±1.40 | 0.12 | 0.372 | 1.06 |
|  | C22:1 | 0.07±0.20 | 0.03±0.10 | 0.44 | 0.605 | -1.28 |
|  | C24:1 | 0.09±0.04 | 0.08±0.03 | 0.35 | 0.636 | -0.14 |
| **ω-6 PUFAs** | C18:2 | 77.53±35.58 | 112.10±50.99 | **1.25** | **0.025** | 1.45 |
|  | C20:2 | 3.07±2.42 | 3.89±2.21 | 0.60 | 0.123 | 1.27 |
|  | C20:3 | 0.86±0.45 | 1.41±0.95 | **1.17** | **0.011** | **1.64** |
|  | C20:4 | 2.53±1.35 | 3.51±1.51 | **1.11** | **0.033** | 1.39 |
|  | C22:2 | 0 | 0 | 0 | 1 | 0 |
| **ω-3 PUFAs** | C18:3 | 1.39±0.66 | 1.71±0.71 | 0.76 | 0.152 | 1.23 |
|  | C20:5 | 0.35±0.20 | 0.61±0.47 | **1.15** | 0.060 | 1.74 |
|  | C22:6 | 2.00±0.86 | 2.78±1.19 | **1.20** | **0.023** | 1.39 |
| **OCFAs** | C7:0 | 0.18±0.225 | 0.55±0.39 | **1.66** | **0.001** | **3.00** |
|  | C9:0 | 2.05±4.16 | 6.74±5.67 | **1.45** | **0.002** | **3.29** |
|  | C11:0 | 0.06±0.11 | 0.20±0.16 | **1.59** | **0.001** | **3.41** |
|  | C13:0 | 0.05±0.04 | 0.08±0.06 | 0.91 | 0.063 | 1.59 |
|  | C15:0 | 0.03±0.04 | 0.05±0.05 | 0.58 | 0.459 | 1.45 |
|  | C17:0 | 0.26±0.28 | 0.36±0.25 | 0.60 | 0.074 | 1.36 |
|  | C19:0 | 0.03±0.04 | 0.04±0.04 | 0.27 | 0.825 | 1.19 |
|  | C21:0 | 0 | 0 | 0 | 1 | 0 |
|  | C23:0 | 0 | 0.09±0.42 | 0.53 | 0.317 | 0 |

Abbreviations: SFA, saturated fatty acid; MUFA, monounsaturated fatty acid; ω-6 PUFA, ω-6 polyunsaturated fatty acid; ω-3 PUFA, ω-3 polyunsaturated fatty acid; OCFA, odd-chain fatty acid; VIP, Variable importance in the projection; FC, fold change.

a The variable importance in the projection (VIP) was obtained in the OPLS-DA. The values in boldface indicate VIP>1.

b The *P*-values were calculated from the nonparametric Mann-Whitney U test. The values in boldface indicate P<0.05.

c The fold changes (FCs) were calculated from the intra-group means of the FFA levels, with a positive value indicating a relatively higher concentration in the OB group and a negative value indicating a relatively lower concentration compared with the NW group. The numbers in boldface indicate that the absolute FC value is >1.5.

**Table S5.** 34 FFAs detected by UHPLC-MS and analyzed by OPLS-DA and the Mann-Whitney U-test in NW and OB group of Han males.

| **category** | **Free fatty acid** | **Mean±standard deviation**  **NW (10) OB (10)** | | ***VIP^a^*** | ***P* Value^b^** | ***FC^c^*** |
| --- | --- | --- | --- | --- | --- | --- |
| **SFAs** | C8:0 | 0.61±0.34 | 1.06±0.82 | **1.07** | 0.199 | **1.73** |
|  | C10:0 | 0.24±0.21 | 0.43±0.36 | 0.96 | 0.131 | **1.77** |
|  | C12:0 | 0.37±0.18 | 0.64±0.27 | **1.43** | **0.023** | **1.72** |
|  | C14:0 | 3.83±2.23 | 7.18±2.90 | **1.51** | **0.013** | **1.88** |
|  | C16:0 | 714.07±353.60 | 934.79±683.69 | 0.58 | 0.496 | 1.31 |
|  | C18:0 | 0 | 0 | 0 | 1 | 0 |
|  | C20:0 | 0.25±0.25 | 0.25±0.15 | 0.05 | 0.597 | -0.02 |
|  | C22:0 | 0 | 0 | 0 | 1 | 0 |
|  | C24:0 | 0.56±0.35 | 0.54±0.17 | 0.12 | 0.545 | -0.06 |
| **MUFAs** | C14:1 | 1.12±1.88 | 1.19±2.47 | 0.03 | 0.723 | 1.06 |
|  | C15:1 | 0 | 0 | 0 | 1 | 0 |
|  | C16:1 | 14.02±12.94 | 16.33±8.81 | 0.30 | 0.290 | 1.16 |
|  | C17:1 | 0.13±0.11 | 0.20±0.10 | 0.91 | 0.131 | **1.55** |
|  | C18:1 | 103.05±81.03 | 157.32±96.06 | 0.89 | 0.174 | **1.53** |
|  | C20:1 | 1.94±2.07 | 1.54±0.73 | 0.37 | 0.545 | -0.33 |
|  | C22:1 | 0.14±0.27 | 0 | **1.08** | 0.068 | **-8.13** |
|  | C24:1 | 0.10±0.06 | 0.08±0.03 | 0.48 | 0.762 | -0.24 |
| **ω-6 PUFAs** | C18:2 | 69.45±33.15 | 125.66±62.21 | **1.50** | **0.016** | **1.81** |
|  | C20:2 | 2.54±1.75 | 4.46±2.89 | **1.09** | 0.096 | **1.76** |
|  | C20:3 | 0.73±0.23 | 1.64±1.22 | **1.42** | **0.013** | **2.25** |
|  | C20:4 | 2.25±1.11 | 3.99±1.34 | **1.59** | **0.008** | **1.77** |
|  | C22:2 | 0 | 0 | 0 | 1 | 0 |
| **ω-3 PUFAs** | C18:3 | 1.27±0.52 | 1.91±0.79 | **1.23** | 0.059 | **1.51** |
|  | C20:5 | 0.39±0.24 | 0.59±0.42 | 0.76 | 0.326 | **1.52** |
|  | C22:6 | 1.97±0.81 | 2.98±1.30 | **1.21** | **0.049** | **1.51** |
| **OCFAs** | C7:0 | 0.18±0.31 | 0.52±0.48 | **1.16** | 0.051 | **2.84** |
|  | C9:0 | 2.75±5.62 | 6.21±6.73 | 0.81 | 0.061 | **2.26** |
|  | C11:0 | 0.07±0.14 | 0.17±0.18 | 0.84 | 0.136 | **2.30** |
|  | C13:0 | 0.05±0.05 | 0.08±0.07 | 0.60 | 0.354 | 1.47 |
|  | C15:0 | 0.04±0.03 | 0.07±0.06 | **1.01** | 0.160 | **1.87** |
|  | C17:0 | 0.28±0.27 | 0.46±0.26 | 0.89 | 0.131 | 1.63 |
|  | C19:0 | 0.03±0.04 | 0.05±0.05 | 0.65 | 0.354 | **1.59** |
|  | C21:0 | 0 | 0 | 0 | 1 | 0 |
|  | C23:0 | 0 | 0.19±0.60 | 0.71 | 0.317 | 0 |

Abbreviations: SFA, saturated fatty acid; MUFA, monounsaturated fatty acid; ω-6 PUFA, ω-6 polyunsaturated fatty acid; ω-3 PUFA, ω-3 polyunsaturated fatty acid; OCFA, odd-chain fatty acid; VIP, Variable importance in the projection; FC, fold change.

a The variable importance in the projection (VIP) was obtained in the OPLS-DA. The values in boldface indicate VIP>1.

b The *P*-values were calculated from the nonparametric Mann-Whitney U test. The values in boldface indicate P<0.05.

c The fold changes (FCs) were calculated from the intra-group means of the FFA levels, with a positive value indicating a relatively higher concentration in the OB group and a negative value indicating a relatively lower concentration compared with the NW group. The numbers in boldface indicate that the absolute FC value is >1.5.

**Table S6.** 34 FFAs detected by UHPLC-MS and analyzed by OPLS-DA and the Mann-Whitney U-test in NW and OB group of Han females.

| **category** | **Free fatty acid** | **Mean±standard deviation**  **NW (10) OB (10)** | | ***VIP^a^*** | ***P* Value^b^** | ***FC^c^*** |
| --- | --- | --- | --- | --- | --- | --- |
| **SFAs** | C8:0 | 0.47±0.15 | 0.75±0.31 | **1.30** | **0.049** | **1.61** |
|  | C10:0 | 0.18±0.09 | 0.31±0.15 | **1.24** | **0.049** | **1.69** |
|  | C12:0 | 0.58±0.25 | 0.47±0.24 | 0.79 | 0.450 | -0.29 |
|  | C14:0 | 4.45±3.66 | 5.25±2.65 | 0.48 | 0.290 | 1.18 |
|  | C16:0 | 718.12±368.17 | 1023.5±508.90 | **1.02** | 0.174 | 1.43 |
|  | C18:0 | 0 | 0 | 0 | 1 | 0 |
|  | C20:0 | 0.16±0.09 | 0.19±0.13 | 0.24 | 0.677 | 1.17 |
|  | C22:0 | 0 | 0 | 0 | 1 | 0 |
|  | C24:0 | 0.40±0.20 | 0.35±0.19 | 0.48 | 0.326 | -0.23 |
| **MUFAs** | C14:1 | 2.28±4.66 | 1.50±2.37 | 0.35 | 0.639 | -0.61 |
|  | C15:1 | 0 | 0 | 0 | 1 | 0 |
|  | C16:1 | 11.55±11.366 | 13.42±5.74 | 0.35 | 0.326 | 1.16 |
|  | C17:1 | 0.12±0.12 | 0.12±0.08 | 0.09 | 0.364 | 1.05 |
|  | C18:1 | 110.74±69.96 | 136.96±62.62 | 0.53 | 0.364 | 1.24 |
|  | C20:1 | 1.21±0.87 | 1.80±1.90 | 0.71 | 0.545 | 1.49 |
|  | C22:1 | 0 | 0.06±0.14 | 0.81 | 0.147 | 0 |
|  | C24:1 | 0.07±0.02 | 0.07±0.03 | 0.07 | 0.426 | -0.01 |
| **ω-6 PUFAs** | C18:2 | 85.62±37.78 | 98.55±34.81 | 0.58 | 0.406 | 1.15 |
|  | C20:2 | 3.60±2.95 | 3.32±1.14 | 0.25 | 0.762 | -0.11 |
|  | C20:3 | 0.99±0.59 | 1.18±0.53 | 0.51 | 0.364 | 1.19 |
|  | C20:4 | 2.81±1.57 | 3.04±1.58 | 0.24 | 0.762 | 1.08 |
|  | C22:2 | 0 | 0 | 0 | 1 | 0 |
| **ω-3 PUFAs** | C18:3 | 1.52±0.79 | 1.50±0.59 | 0.03 | 0.966 | -0.01 |
|  | C20:5 | 0.31±0.15 | 0.63±0.54 | **1.35** | 0.131 | **2.02** |
|  | C22:6 | 2.03±0.94 | 2.58±1.11 | 0.89 | 0.174 | 1.27 |
| **OCFAs** | C7:0 | 0.18±0.18 | 0.57±0.30 | **1.99** | **0.003** | **3.15** |
|  | C9:0 | 1.35±1.98 | 7.26±4.69 | **2.05** | **0.006** | **5.37** |
|  | C11:0 | 0.04±0.07 | 0.24±0.14 | **2.23** | **0.001** | **5.17** |
|  | C13:0 | 0.05±0.04 | 0.08±0.06 | **1.11** | 0.085 | 1.72 |
|  | C15:0 | 0.03±0.04 | 0.03±0.04 | 0.03 | 0.668 | -0.13 |
|  | C17:0 | 0.24±0.30 | 0.26±0.21 | 0.09 | 0.364 | 1.06 |
|  | C19:0 | 0.03±0.03 | 0.03±0.03 | 0.40 | 0.562 | -0.35 |
|  | C21:0 | 0 | 0 | 0 | 1 | 0 |
|  | C23:0 | 0 | 0 | 0 | 1 | 0 |

Abbreviations: SFA, saturated fatty acid; MUFA, monounsaturated fatty acid; ω-6 PUFA, ω-6 polyunsaturated fatty acid; ω-3 PUFA, ω-3 polyunsaturated fatty acid; OCFA, odd-chain fatty acid; VIP, Variable importance in the projection; FC, fold change.

a The variable importance in the projection (VIP) was obtained in the OPLS-DA. The values in boldface indicate VIP>1.

b The *P*-values were calculated from the nonparametric Mann-Whitney U test. The values in boldface indicate P<0.05.

c The fold changes (FCs) were calculated from the intra-group means of the FFA levels, with a positive value indicating a relatively higher concentration in the OB group and a negative value indicating a relatively lower concentration compared with the NW group. The numbers in boldface indicate that the absolute FC value is >1.5.

**Table S7.** Significant differences in FFAs detected by UHPLC-MS and analyzed by OPLS-DA and Mann-Whitney U test between the male and female groups in NW group of Han nationality.

| **category** | **Free fatty acid** | **Normal Weight(NW)**  **Male(10) Female(10)** | | ***VIP^a^*** | ***P* Value^b^** | ***FC^c^*** |
| --- | --- | --- | --- | --- | --- | --- |
| **SFAs** | C8:0 | 0.61±0.34 | 0.47±0.15 | 0.79 | 0.369 | -1.32 |
|  | C10:0 | 0.24±0.21 | 0.18±0.09 | 0.65 | 0.364 | -1.34 |
|  | C12:0 | 0.37±0.18 | 0.58±0.25 | **1.61** | 0.059 | **1.57** |
|  | C14:0 | 3.83±2.23 | 4.45±3.66 | 0.39 | 0.940 | 1.16 |
|  | C16:0 | 714.07±353.60 | 718.12±368.17 | 0.06 | 0.762 | 1.01 |
|  | C18:0 | 0 | 0 | 0 | 1 | 0 |
|  | C20:0 | 0.25±0.25 | 0.16±0.09 | 0.95 | 0.597 | **-1.55** |
|  | C22:0 | 0 | 0 | 0 | 1 | 0 |
|  | C24:0 | 0.56±0.35 | 0.40±0.20 | **1.25** | 0.253 | -1.38 |
| **MUFAs** | C14:1 | 1.12±1.88 | 2.28±4.66 | 0.72 | 0.650 | **2.04** |
|  | C15:1 | 0 | 0 | 0 | 1 | 0 |
|  | C16:1 | 14.02±12.94 | 11.55±11.366 | 0.45 | 0.650 | -1.21 |
|  | C17:1 | 0.13±0.11 | 0.12±0.12 | 0.19 | 0.880 | -1.11 |
|  | C18:1 | 103.05±81.03 | 110.74±69.96 | 0.25 | 0.705 | 1.07 |
|  | C20:1 | 1.94±2.07 | 1.21±0.87 | 0.88 | 0.597 | **-1.60** |
|  | C22:1 | 0.14±0.27 | 0 | 1.45 | 1 | 0 |
|  | C24:1 | 0.10±0.06 | 0.07±0.02 | **1.16** | 0.462 | -1.32 |
| **ω-6 PUFAs** | C18:2 | 69.45±33.15 | 85.62±37.78 | 0.78 | 0.450 | 1.23 |
|  | C20:2 | 2.54±1.75 | 3.60±2.95 | 0.90 | 0.545 | 1.42 |
|  | C20:3 | 0.73±0.23 | 0.99±0.59 | 0.84 | 0.545 | 1.36 |
|  | C20:4 | 2.25±1.11 | 2.81±1.57 | 0.74 | 0.290 | 1.25 |
|  | C22:2 | 0 | 0 | 0 | 1 | 0 |
| **ω-3 PUFAs** | C18:3 | 1.27±0.52 | 1.52±0.79 | 0.69 | 0.496 | 1.20 |
|  | C20:5 | 0.39±0.24 | 0.31±0.15 | 0.56 | 0.364 | 1.24 |
|  | C22:6 | 1.97±0.81 | 2.03±0.94 | 0.05 | 1.0 | 1.03 |
| **OCFAs** | C7:0 | 0.18±0.31 | 0.18±0.18 | 0.003 | 0.394 | -1.01 |
|  | C9:0 | 2.75±5.62 | 1.35±1.98 | 0.57 | 0.394 | **2.03** |
|  | C11:0 | 0.07±0.14 | 0.04±0.07 | 0.45 | 0.816 | **-1.67** |
|  | C13:0 | 0.05±0.05 | 0.05±0.04 | 0.25 | 0.817 | -1.03 |
|  | C15:0 | 0.04±0.03 | 0.03±0.04 | 0.47 | 0.191 | 1.29 |
|  | C17:0 | 0.28±0.27 | 0.24±0.30 | 0.22 | 0.650 | -1.14 |
|  | C19:0 | 0.03±0.04 | 0.03±0.03 | 0.003 | 0.870 | -1.05 |
|  | C21:0 | 0 | 0 | 0 | 1 | 0 |
|  | C23:0 | 0 | 0 | 0 | 1 | 0 |

Abbreviations: SFA, saturated fatty acid; MUFA, monounsaturated fatty acid; ω-6 PUFA, ω-6 polyunsaturated fatty acid; ω-3 PUFA, ω-3 polyunsaturated fatty acid; OCFA, odd-chain fatty acid; VIP, Variable importance in the projection; FC, fold change.

a The variable importance in the projection (VIP) was obtained in the OPLS-DA. The values in boldface indicate VIP>1.

b The *P*-values were calculated from the nonparametric Mann-Whitney U test. The values in boldface indicate P<0.05.

c The fold changes (FCs) were calculated from the intra-group means of the FFA levels, with a positive value indicating a relatively higher concentration in the OB group and a negative value indicating a relatively lower concentration compared with the NW group. The numbers in boldface indicate that the absolute FC value is >1.5.

**Table S8.** Significant differences in FFAs detected by UHPLC-MS and analyzed by OPLS-DA and Mann-Whitney U test between the male and female groups in OB group of Han nationality.

| **category** | **Free fatty acid** | **Obese(OB)**  **Male(10) Female(10)** | | ***VIP^a^*** | ***P* Value^b^** | ***FC^c^*** |
| --- | --- | --- | --- | --- | --- | --- |
| **SFAs** | C8:0 | 1.06±0.82 | 0.75±0.31 | 0.96 | 0.545 | -1.42 |
|  | C10:0 | 0.43±0.36 | 0.31±0.15 | 0.85 | 0.705 | -1.41 |
|  | C12:0 | 0.64±0.27 | 0.47±0.24 | **1.09** | 0.174 | -1.35 |
|  | C14:0 | 7.18±2.90 | 5.25±2.65 | **1.05** | 0.199 | -1.37 |
|  | C16:0 | 934.79±683.69 | 1023.5±508.90 | 0.25 | 0.496 | -1.09 |
|  | C18:0 | 0 | 0 | 0 | 1 | 0 |
|  | C20:0 | 0.25±0.15 | 0.19±0.13 | 0.60 | 0.257 | -1.30 |
|  | C22:0 | 0 | 0 | 0 | 1 | 0 |
|  | C24:0 | 0.54±0.17 | 0.35±0.19 | **1.35** | **0.041** | **-1.55** |
| **MUFAs** | C14:1 | 1.19±2.47 | 1.50±2.37 | 0.18 | 0.705 | 1.21 |
|  | C15:1 | 0 | 0 | 0 | 1 | 0 |
|  | C16:1 | 16.33±8.81 | 13.42±5.74 | 0.60 | 0.290 | -1.22 |
|  | C17:1 | 0.20±0.10 | 0.12±0.08 | 1.22 | 0.082 | **-1.64** |
|  | C18:1 | 157.32±96.06 | 136.96±62.62 | 0.41 | 0.762 | -1.15 |
|  | C20:1 | 1.54±0.73 | 1.80±1.90 | 0.26 | 0.496 | 1.14 |
|  | C22:1 | 0 | 0.06±0.14 | 0 | 1 | 0 |
|  | C24:1 | 0.08±0.03 | 0.07±0.03 | 0.46 | 0.364 | -1.12 |
| **ω-6 PUFAs** | C18:2 | 125.66±62.21 | 98.55±34.81 | 0.96 | 0.151 | -1.28 |
|  | C20:2 | 4.46±2.89 | 3.32±1.14 | 0.80 | 0.650 | -1.34 |
|  | C20:3 | 1.64±1.22 | 1.18±0.53 | 0.89 | 0.545 | -1.39 |
|  | C20:4 | 3.99±1.34 | 3.04±1.58 | 1.05 | 0.151 | -1.31 |
|  | C22:2 | 0 | 0 | 0 | 1 | 0 |
| **ω-3 PUFAs** | C18:3 | 1.91±0.79 | 1.50±0.59 | 0.90 | 0.199 | -1.27 |
|  | C20:5 | 0.59±0.42 | 0.63±0.54 | 0.24 | 0.821 | 1.07 |
|  | C22:6 | 2.98±1.30 | 2.58±1.11 | 0.51 | 0.650 | -1.16 |
| **OCFAs** | C7:0 | 0.52±0.48 | 0.57±0.30 | 0.23 | 0.722 | 1.09 |
|  | C9:0 | 6.21±6.73 | 7.26±4.69 | 0.33 | 0.923 | 1.14 |
|  | C11:0 | 0.17±0.18 | 0.24±0.14 | 0.70 | 0.446 | 1.27 |
|  | C13:0 | 0.08±0.07 | 0.08±0.06 | 0.12 | 0.619 | 1.04 |
|  | C15:0 | 0.07±0.06 | 0.03±0.04 | **1.54** | 0.060 | **-2.66** |
|  | C17:0 | 0.46±0.26 | 0.26±0.21 | **1.22** | 0.082 | **-1.75** |
|  | C19:0 | 0.05±0.05 | 0.03±0.03 | **1.10** | 0.208 | **-2.15** |
|  | C21:0 | 0 | 0 | 0 | 1 | 0 |
|  | C23:0 | 0.19±0.60 | 0 | 0 | 0.028 | 0 |

Abbreviations: SFA, saturated fatty acid; MUFA, monounsaturated fatty acid; ω-6 PUFA, ω-6 polyunsaturated fatty acid; ω-3 PUFA, ω-3 polyunsaturated fatty acid; OCFA, odd-chain fatty acid; VIP, Variable importance in the projection; FC, fold change.

a The variable importance in the projection (VIP) was obtained in the OPLS-DA. The values in boldface indicate VIP>1.

b The *P*-values were calculated from the nonparametric Mann-Whitney U test. The values in boldface indicate P<0.05.

c The fold changes (FCs) were calculated from the intra-group means of the FFA levels, with a positive value indicating a relatively higher concentration in the OB group and a negative value indicating a relatively lower concentration compared with the NW group. The numbers in boldface indicate that the absolute FC value is >1.5.

**Table S9.** 34 FFAs detected by UHPLC-MS and analyzed by OPLS-DA and the Mann-Whitney U-test in NW and OB group of Uygur.

| **category** | **Free fatty acid** | **Mean±standard deviation**  **NW (20) OB (20)** | | ***VIP^a^*** | ***P* Value^b^** | ***FC^c^*** |
| --- | --- | --- | --- | --- | --- | --- |
| **SFAs** | C8:0 | 0.66±0.33 | 0.69±0.51 | 0.15 | 0.685 | 1.05 |
|  | C10:0 | 0.29±0.21 | 0.30±0.27 | 0.00 | 0.694 | 1.06 |
|  | C12:0 | 0.40±0.40 | 0.48±0.36 | 0.40 | 0.389 | 1.19 |
|  | C14:0 | 3.49±2.75 | 4.54±3.24 | 0.55 | 0.339 | 1.37 |
|  | C16:0 | 40.54±29.88 | 53.06±35.68 | 0.99 | 0.238 | 1.38 |
|  | C18:0 | 0 | 0 | 0 | 1 | 0 |
|  | C20:0 | 0.62±1.31 | 0.61±1.02 | 0.09 | 0.223 | -0.38 |
|  | C22:0 | 0 | 0 | 0 | 1 | 0 |
|  | C24:0 | 0.36±0.14 | 0.42±0.26 | 0.59 | 0.692 | -0.03 |
| **MUFAs** | C14:1 | 0 | 0 | 0 | 1 | 0 |
|  | C15:1 | 0 | 0 | 0 | 1 | 0 |
|  | C16:1 | 3.68±3.45 | 6.18±5.17 | 0.99 | 0.094 | **1.68** |
|  | C17:1 | 7.76 | 0.40±0.38 | 0.77 | 0.221 | **-3.26** |
|  | C18:1 | 30.37±18.26 | 40.11±24.70 | 0.95 | 0.152 | 1.32 |
|  | C20:1 | 0.16±0.14 | 0.14±0.10 | 0.45 | 0.934 | 1.15 |
|  | C22:1 | 0 | 0 | 0 | 1 | 0 |
|  | C24:1 | 0.12±0.04 | 0.11±0.06 | 0.25 | 0.559 | -0.28 |
| **ω-6 PUFAs** | C18:2 | 37.58±26.56 | 47.42±27.99 | 0.90 | 0.152 | 1.26 |
|  | C20:2 | 0.59±0.68 | 0.71±0.65 | 0.44 | 0.279 | 1.19 |
|  | C20:3 | 0.88±0.64 | 1.22±0.71 | **1.24** | **0.023** | 1.39 |
|  | C20:4 | 1.81±0.82 | 2.24±1.13 | **1.01** | 0.291 | 1.24 |
|  | C22:2 | 0.03±0.03 | 0.01±0.00 | 0.42 | 0.521 | **-2.18** |
| **ω-3 PUFAs** | C18:3 | 0.51±0.26 | 0.81±0.55 | **1.60** | 0.062 | **1.59** |
|  | C20:5 | 0.18±0.11 | 0.31±0.22 | **1.04** | **0.020** | **1.76** |
|  | C22:6 | 1.17±0.77 | 1.36±0.83 | 0.52 | 0.482 | 1.16 |
| **OCFAs** | C7:0 | 0.11±0.07 | 0.11±0.08 | 0.06 | 0.978 | -0.63 |
|  | C9:0 | 0.11±0.08 | 0.17±0.08 | **1.03** | 0.076 | **1.55** |
|  | C11:0 | 0.01±0.01 | 0.02±0.01 | 0.69 | 0.205 | 1.30 |
|  | C13:0 | 0.02±0.02 | 0.03±0.01 | 0.01 | 0.414 | 1.19 |
|  | C15:0 | 0.40±0.29 | 0.49±0.35 | 0.55 | 0.384 | 1.30 |
|  | C17:0 | 1.02±0.68 | 1.25±0.82 | 0.67 | 0.279 | 1.23 |
|  | C19:0 | 0.04±0.05 | 0.07±0.05 | **1.18** | 0.078 | 1.17 |
|  | C21:0 | 0 | 0 | 0 | 1 | 0 |
|  | C23:0 | 0 | 0 | 0 | 1 | 0 |

Abbreviations: SFA, saturated fatty acid; MUFA, monounsaturated fatty acid; ω-6 PUFA, ω-6 polyunsaturated fatty acid; ω-3 PUFA, ω-3 polyunsaturated fatty acid; OCFA, odd-chain fatty acid; VIP, Variable importance in the projection; FC, fold change.

a The variable importance in the projection (VIP) was obtained in the OPLS-DA. The values in boldface indicate VIP>1.

b The *P*-values were calculated from the nonparametric Mann-Whitney U test. The values in boldface indicate P<0.05.

c The fold changes (FCs) were calculated from the intra-group means of the FFA levels, with a positive value indicating a relatively higher concentration in the OB group and a negative value indicating a relatively lower concentration compared with the NW group. The numbers in boldface indicate that the absolute FC value is >1.5.

**Table S10.** 34 FFAs detected by UHPLC-MS and analyzed by OPLS-DA and the Mann-Whitney U-test in NW and OB group of Uygur males.

| **category** | **Free fatty acid** | **Mean±standard deviation**  **NW (10) OB (10)** | | ***VIP^a^*** | ***P* Value^b^** | ***FC^c^*** |
| --- | --- | --- | --- | --- | --- | --- |
| **SFAs** | C8:0 | 0.81±0.36 | 0.74±0.67 | 0.26 | 0.290 | -0.12 |
|  | C10:0 | 0.40±0.23 | 0.32±0.36 | 0.73 | 0.165 | -0.18 |
|  | C12:0 | 0.54±0.51 | 0.43±0.34 | 0.57 | 0.691 | -0.35 |
|  | C14:0 | 4.38±3.14 | 3.48±2.62 | 0.76 | 0.462 | -0.18 |
|  | C16:0 | 43.29±31.83 | 41.78±33.68 | 0.14 | 0.935 | -0.17 |
|  | C18:0 | 0 | 0 | 0 | 1 | 0 |
|  | C20:0 | 1.43±2.25 | 0.21±0.07 | 0.27 | 0.564 | **-3.37** |
|  | C22:0 | 0 | 0 | 0 | 1 | 0 |
|  | C24:0 | 0.38±0.17 | 0.38±0.21 | 0.09 | 0.790 | -0.35 |
| **MUFAs** | C14:1 | 0 | 0 | 0 | 1 | 0 |
|  | C15:1 | 0 | 0 | 0 | 1 | 0 |
|  | C16:1 | 3.61±4.13 | 3.90±2.70 | 0.15 | 0.406 | 1.08 |
|  | C17:1 | 7.76 | 0.67 | **1.11** | 0.317 | **-3.53** |
|  | C18:1 | 28.45±19.01 | 34.05±19.13 | 0.62 | 0.364 | 1.20 |
|  | C20:1 | 0.16±0.20 | 0.18±0.03 | 0.02 | 0.480 | 1.47 |
|  | C22:1 | 0 | 0 | 0 | 1 | 0 |
|  | C24:1 | 0.12±0.06 | 0.12±0.06 | 0.15 | 0.870 | -0.13 |
| **ω-6 PUFAs** | C18:2 | 37.80±30.00 | 39.75±26.92 | 0.18 | 0.630 | 1.05 |
|  | C20:2 | 0.58±0.84 | 0.58±0.43 | 0.02 | 0.199 | -0.02 |
|  | C20:3 | 0.90±0.80 | 1.13±0.53 | 0.92 | 0.174 | 1.26 |
|  | C20:4 | 1.68±0.74 | 2.07±0.80 | **1.16** | 0.257 | 1.23 |
|  | C22:2 | 0.05 | 0 | 0.63 | 1 | 0 |
| **ω-3 PUFAs** | C18:3 | 0.46±0.22 | 0.56±0.21 | 0.82 | 0.199 | 1.22 |
|  | C20:5 | 0.14±0.10 | 0.30±0.27 | **1.22** | 0.131 | **2.08** |
|  | C22:6 | 0.91±0.56 | 1.27±0.78 | **1.17** | 0.226 | 1.39 |
| **OCFAs** | C7:0 | 0.13±0.08 | 0.07±0.08 | **1.19** | 0.372 | -1.31 |
|  | C9:0 | 0.16±0.08 | 0.18±0.11 | 0.04 | 0.571 | 1.13 |
|  | C11:0 | 0.01±0.01 | 0.02±0.01 | 0.96 | 0.345 | 1.18 |
|  | C13:0 | 0.03±0.02 | 0.03±0.02 | 0.08 | 0.917 | -0.05 |
|  | C15:0 | 0.47±0.33 | 0.49±0.38 | 0.29 | 0.911 | 1.16 |
|  | C17:0 | 1.04±0.76 | 1.34±0.97 | 0.88 | 0.406 | 1.29 |
|  | C19:0 | 0.06±0.06 | 0.05±0.05 | **1.18** | 0.817 | -0.41 |
|  | C21:0 | 0 | 0 | 0 | 1 | 0 |
|  | C23:0 | 0 | 0 | 0 | 1 | 0 |

Abbreviations: SFA, saturated fatty acid; MUFA, monounsaturated fatty acid; ω-6 PUFA, ω-6 polyunsaturated fatty acid; ω-3 PUFA, ω-3 polyunsaturated fatty acid; OCFA, odd-chain fatty acid; VIP, Variable importance in the projection; FC, fold change.

a The variable importance in the projection (VIP) was obtained in the OPLS-DA. The values in boldface indicate VIP>1.

b The *P*-values were calculated from the nonparametric Mann-Whitney U test. The values in boldface indicate P<0.05.

c The fold changes (FCs) were calculated from the intra-group means of the FFA levels, with a positive value indicating a relatively higher concentration in the OB group and a negative value indicating a relatively lower concentration compared with the NW group. The numbers in boldface indicate that the absolute FC value is >1.5.

**Table S11.** 34 FFAs detected by UHPLC-MS and analyzed by OPLS-DA and the Mann-Whitney U-test in NW and OB group of Uygur females.

| **category** | **Free fatty acid** | **Mean±standard deviation**  **NW (10) OB (10)** | | ***VIP^a^*** | ***P* Value^b^** | ***FC^c^*** |
| --- | --- | --- | --- | --- | --- | --- |
| **SFAs** | C8:0 | 0.52±0.23 | 0.65±0.31 | 0.48 | 0.450 | 1.25 |
|  | C10:0 | 0.19±0.13 | 0.27±0.17 | 0.73 | 0.364 | 1.40 |
|  | C12:0 | 0.28±0.22 | 0.53±0.39 | **1.08** | 0.151 | **1.90** |
|  | C14:0 | 2.70±2.20 | 5.60±3.57 | **1.29** | 0.070 | **2.08** |
|  | C16:0 | 38.07±29.51 | 64.34±35.63 | **1.37** | 0.082 | **1.69** |
|  | C18:0 | 0 | 0 | 0 | 1 | 0 |
|  | C20:0 | 0.22±0.38 | 0.78±1.20 | 0.65 | 0.201 | **2.96** |
|  | C22:0 | 0 | 0 | 0 | 1 | 0 |
|  | C24:0 | 0.34±0.11 | 0.46±0.30 | 0.87 | 0.462 | 1.20 |
| **MUFAs** | C14:1 | 0 | 0 | 0 | 1 | 0 |
|  | C15:1 | 0 | 0 | 0 | 1 | 0 |
|  | C16:1 | 3.75±2.83 | 8.47±6.13 | **1.28** | 0.070 | **2.25** |
|  | C17:1 | 0 | 0.14 | 0.37 | 1 | 0 |
|  | C18:1 | 32.29±18.30 | 46.17±28.99 | 0.95 | 0.257 | 1.43 |
|  | C20:1 | 0.17±0.12 | 0.11±0.12 | 0.54 | 0.465 | -0.05 |
|  | C22:1 | 0 | 0 | 0 | 1 | 0 |
|  | C24:1 | 0.11±0.03 | 0.09±0.05 | 0.53 | 0.414 | -0.45 |
| **ω-6 PUFAs** | C18:2 | 37.36±24.27 | 55.10±28.25 | **1.18** | 0.112 | 1.48 |
|  | C20:2 | 0.61±0.52 | 0.84±0.82 | 0.67 | 0.650 | 1.38 |
|  | C20:3 | 0.86±0.47 | 1.30±0.88 | **1.18** | 0.151 | **1.52** |
|  | C20:4 | 1.94±0.92 | 2.40±1.41 | 0.74 | 0.762 | 1.24 |
|  | C22:2 | 0 | 0 | 0 | 1 | 0 |
| **ω-3 PUFAs** | C18:3 | 0.56±0.30 | 1.06±0.67 | **1.78** | 0.131 | **1.90** |
|  | C20:5 | 0.21±0.12 | 0.32±0.17 | 0.71 | 0.096 | **1.54** |
|  | C22:6 | 1.43±0.89 | 1.46±0.91 | 0.04 | 0.821 | 1.02 |
| **OCFAs** | C7:0 | 0.10±0.05 | 0.14±0.07 | 0.82 | 0.143 | -0.10 |
|  | C9:0 | 0.07±0.04 | 0.16±0.06 | **1.55** | **0.014** | **1.78** |
|  | C11:0 | 0.01±0.01 | 0.02±0.01 | 0.37 | 0.522 | **2.15** |
|  | C13:0 | 0.02±0.01 | 0.02±0.01 | 0.12 | 0.327 | 1.66 |
|  | C15:0 | 0.34±0.26 | 0.50±0.33 | 0.67 | 0.174 | 1.49 |
|  | C17:0 | 0.10±0.63 | 1.16±0.68 | 0.37 | 0.406 | 1.16 |
|  | C19:0 | 0.03±0.03 | 0.09±0.04 | **1.97** | **0.009** | **1.94** |
|  | C21:0 | 0 | 0 | 0 | 1 | 0 |
|  | C23:0 | 0 | 0 | 0 | 1 | 0 |

Abbreviations: SFA, saturated fatty acid; MUFA, monounsaturated fatty acid; ω-6 PUFA, ω-6 polyunsaturated fatty acid; ω-3 PUFA, ω-3 polyunsaturated fatty acid; OCFA, odd-chain fatty acid; VIP, Variable importance in the projection; FC, fold change.

a The variable importance in the projection (VIP) was obtained in the OPLS-DA. The values in boldface indicate VIP>1.

b The *P*-values were calculated from the nonparametric Mann-Whitney U test. The values in boldface indicate P<0.05.

c The fold changes (FCs) were calculated from the intra-group means of the FFA levels, with a positive value indicating a relatively higher concentration in the OB group and a negative value indicating a relatively lower concentration compared with the NW group. The numbers in boldface indicate that the absolute FC value is >1.5.

**Table S12.** Significant differences in FFAs detected by UHPLC-MS and analyzed by OPLS-DA and Mann-Whitney U test between the male and female groups in NW group of Uyghur nationality.

| **category** | **Free fatty acid** | **Normal Weight(NW)**  **Male(10) Female(10)** | | ***VIP^a^*** | ***P* Value^b^** | ***FC^c^*** |
| --- | --- | --- | --- | --- | --- | --- |
| **SFAs** | C8:0 | 0.81±0.36 | 0.52±0.23 | **1.09** | **0.041** | **-1.57** |
|  | C10:0 | 0.40±0.23 | 0.19±0.13 | **1.72** | **0.034** | **-2.06** |
|  | C12:0 | 0.54±0.51 | 0.28±0.22 | **1.16** | 0.142 | **-1.94** |
|  | C14:0 | 4.38±3.14 | 2.70±2.20 | 0.99 | 0.496 | **-1.62** |
|  | C16:0 | 43.29±31.83 | 38.07±29.51 | 0.11 | 0.880 | -1.14 |
|  | C18:0 | 0 | 0 | 0 | 1.0 | 0 |
|  | C20:0 | 1.43±2.25 | 0.22±0.38 | 0.96 | 0.831 | **-6.52** |
|  | C22:0 | 0 | 0 | 0 | 1.0 | 0 |
|  | C24:0 | 0.38±0.17 | 0.34±0.11 | 0.35 | 0.597 | -1.12 |
| **MUFAs** | C14:1 | 0 | 0 | 0 | 1.0 | 0 |
|  | C15:1 | 0 | 0 | 0 | 1.0 | 0 |
|  | C16:1 | 3.61±4.13 | 3.75±2.83 | 0.06 | 0.650 | 1.04 |
|  | C17:1 | 7.76 | 0 | 0 | 0.450 | 0 |
|  | C18:1 | 28.45±19.01 | 32.29±18.30 | 0.36 | 0.650 | 1.12 |
|  | C20:1 | 0.16±0.20 | 0.17±0.12 | 0.26 | 0.821 | 1.04 |
|  | C22:1 | 0 | 0 | 0 | 1.0 | 0 |
|  | C24:1 | 0.12±0.06 | 0.11±0.03 | 0.03 | 0.762 | -1.02 |
| **ω-6 PUFAs** | C18:2 | 37.80±30.00 | 37.36±24.27 | 0.02 | 1.0 | -1.01 |
|  | C20:2 | 0.58±0.84 | 0.61±0.52 | 0.08 | 0.450 | 1.04 |
|  | C20:3 | 0.90±0.80 | 0.86±0.47 | 0.15 | 0.545 | -1.05 |
|  | C20:4 | 1.68±0.74 | 1.94±0.92 | 0.44 | 0.364 | 1.23 |
|  | C22:2 | 0.05 | 0 | 0 | 0.386 | 0 |
| **ω-3 PUFAs** | C18:3 | 0.46±0.22 | 0.56±0.30 | 0.52 | 0.545 | 1.18 |
|  | C20:5 | 0.14±0.10 | 0.21±0.12 | 0.46 | 0.257 | 1.30 |
|  | C22:6 | 0.91±0.56 | 1.43±0.89 | **1.18** | 0.151 | 1.36 |
| **OCFAs** | C7:0 | 0.13±0.08 | 0.10±0.05 | 0.52 | 0.418 | -1.35 |
|  | C9:0 | 0.16±0.08 | 0.07±0.04 | **1.58** | **0.013** | **-2.39** |
|  | C11:0 | 0.01±0.01 | 0.01±0.01 | 0.12 | 0.317 | -1.14 |
|  | C13:0 | 0.03±0.02 | 0.02±0.01 | 0.57 | 0.290 | **-1.69** |
|  | C15:0 | 0.47±0.33 | 0.34±0.26 | 0.50 | 0.414 | -1.39 |
|  | C17:0 | 1.04±0.76 | 0.10±0.63 | 0.10 | 1.0 | -1.04 |
|  | C19:0 | 0.06±0.06 | 0.03±0.03 | **1.54** | 0.597 | **-2.35** |
|  | C21:0 | 0 | 0 | 0 | 1 | 0 |
|  | C23:0 | 0 | 0 | 0 | 1 | 0 |

Abbreviations: SFA, saturated fatty acid; MUFA, monounsaturated fatty acid; ω-6 PUFA, ω-6 polyunsaturated fatty acid; ω-3 PUFA, ω-3 polyunsaturated fatty acid; OCFA, odd-chain fatty acid; VIP, Variable importance in the projection; FC, fold change.

a The variable importance in the projection (VIP) was obtained in the OPLS-DA. The values in boldface indicate VIP>1.

b The *P*-values were calculated from the nonparametric Mann-Whitney U test. The values in boldface indicate P<0.05.

c The fold changes (FCs) were calculated from the intra-group means of the FFA levels, with a positive value indicating a relatively higher concentration in the OB group and a negative value indicating a relatively lower concentration compared with the NW group. The numbers in boldface indicate that the absolute FC value is >1.5

**Table S13.** Significant differences in FFAs detected by UHPLC-MS and analyzed by OPLS-DA and Mann-Whitney U test between the male and female groups in the OB group of Uygur nationality.

| **category** | **Free fatty acid** | **Normal Weight(NW)**  **Male(10) Female(10)** | | ***VIP^a^*** | ***P* Value^b^** | ***FC^c^*** |
| --- | --- | --- | --- | --- | --- | --- |
| **SFAs** | C8:0 | 0.74±0.67 | 0.65±0.31 | 0.29 | 1.0 | -1.15 |
|  | C10:0 | 0.32±0.36 | 0.27±0.17 | 0.25 | 0.762 | -1.17 |
|  | C12:0 | 0.43±0.34 | 0.53±0.39 | 0.36 | 0.290 | 1.20 |
|  | C14:0 | 3.48±2.62 | 5.60±3.57 | 0.94 | 0.131 | 1.38 |
|  | C16:0 | 41.78±33.68 | 64.34±35.63 | 0.90 | 0.096 | 1.35 |
|  | C18:0 | 0 | 0 | 0 | 1.0 | 0 |
|  | C20:0 | 0.21±0.07 | 0.78±1.20 | 0.46 | 0.423 | **3.71** |
|  | C22:0 | 0 | 0 | 0 | 1.0 | 0 |
|  | C24:0 | 0.38±0.21 | 0.46±0.30 | 0.43 | 0.354 | 1.18 |
| **MUFAs** | C14:1 | 0 | 0 | 0 | 1.0 | 0 |
|  | C15:1 | 0 | 0 | 0 | 1.0 | 0 |
|  | C16:1 | 3.90±2.70 | 8.47±6.13 | **1.28** | 0.082 | **2.17** |
|  | C17:1 | 0.67 | 0.14 | 0 | 0.450 | **-4.94** |
|  | C18:1 | 34.05±19.13 | 46.17±28.99 | 0.69 | 0.450 | 1.26 |
|  | C20:1 | 0.18±0.03 | 0.11±0.12 | 0.98 | 0.762 | **-1.54** |
|  | C22:1 | 0 | 0 | 0 | 1.0 | 0 |
|  | C24:1 | 0.12±0.06 | 0.09±0.05 | 0.63 | 0.310 | -1.27 |
| **ω-6 PUFAs** | C18:2 | 39.75±26.92 | 55.10±28.25 | 0.80 | 0.131 | 1.28 |
|  | C20:2 | 0.58±0.43 | 0.84±0.82 | 0.61 | 0.597 | 1.28 |
|  | C20:3 | 1.13±0.53 | 1.30±0.88 | 0.39 | 0.821 | 1.31 |
|  | C20:4 | 2.07±0.80 | 2.40±1.41 | 0.46 | 0.940 | 1.13 |
|  | C22:2 | 0 | 0 | 0 | 0.606 | 0 |
| **ω-3 PUFAs** | C18:3 | 0.56±0.21 | 1.06±0.67 | **1.35** | 0.112 | **1.89** |
|  | C20:5 | 0.30±0.27 | 0.32±0.17 | 0.12 | 0.597 | 1.05 |
|  | C22:6 | 1.27±0.78 | 1.46±0.91 | 0.31 | 0.545 | 1.13 |
| **OCFAs** | C7:0 | 0.07±0.08 | 0.14±0.07 | 0.93 | 1.0 | **2.00** |
|  | C9:0 | 0.18±0.11 | 0.16±0.06 | 0.07 | 0.643 | -1.11 |
|  | C11:0 | 0.02±0.01 | 0.02±0.01 | 0.19 | 0.855 | -1.2 |
|  | C13:0 | 0.03±0.02 | 0.02±0.01 | 0.68 | 0.773 | -1.23 |
|  | C15:0 | 0.49±0.38 | 0.50±0.33 | 0.03 | 1.0 | 1.03 |
|  | C17:0 | 1.34±0.97 | 1.16±0.68 | 0.36 | 1.0 | -1.16 |
|  | C19:0 | 0.05±0.05 | 0.09±0.04 | 0.82 | 0.683 | **1.8** |
|  | C21:0 | 0 | 0 | 0 | 1 | 0 |
|  | C23:0 | 0 | 0 | 0 | 1 | 0 |

Abbreviations: SFA, saturated fatty acid; MUFA, monounsaturated fatty acid; ω-6 PUFA, ω-6 polyunsaturated fatty acid; ω-3 PUFA, ω-3 polyunsaturated fatty acid; OCFA, odd-chain fatty acid; VIP, Variable importance in the projection; FC, fold change.

a The variable importance in the projection (VIP) was obtained in the OPLS-DA. The values in boldface indicate VIP>1.

b The *P*-values were calculated from the nonparametric Mann-Whitney U test. The values in boldface indicate P<0.05.

c The fold changes (FCs) were calculated from the intra-group means of the FFA levels, with a positive value indicating a relatively higher concentration in the OB group and a negative value indicating a relatively lower concentration compared with the NW group. The numbers in boldface indicate that the absolute FC value is >1.5.


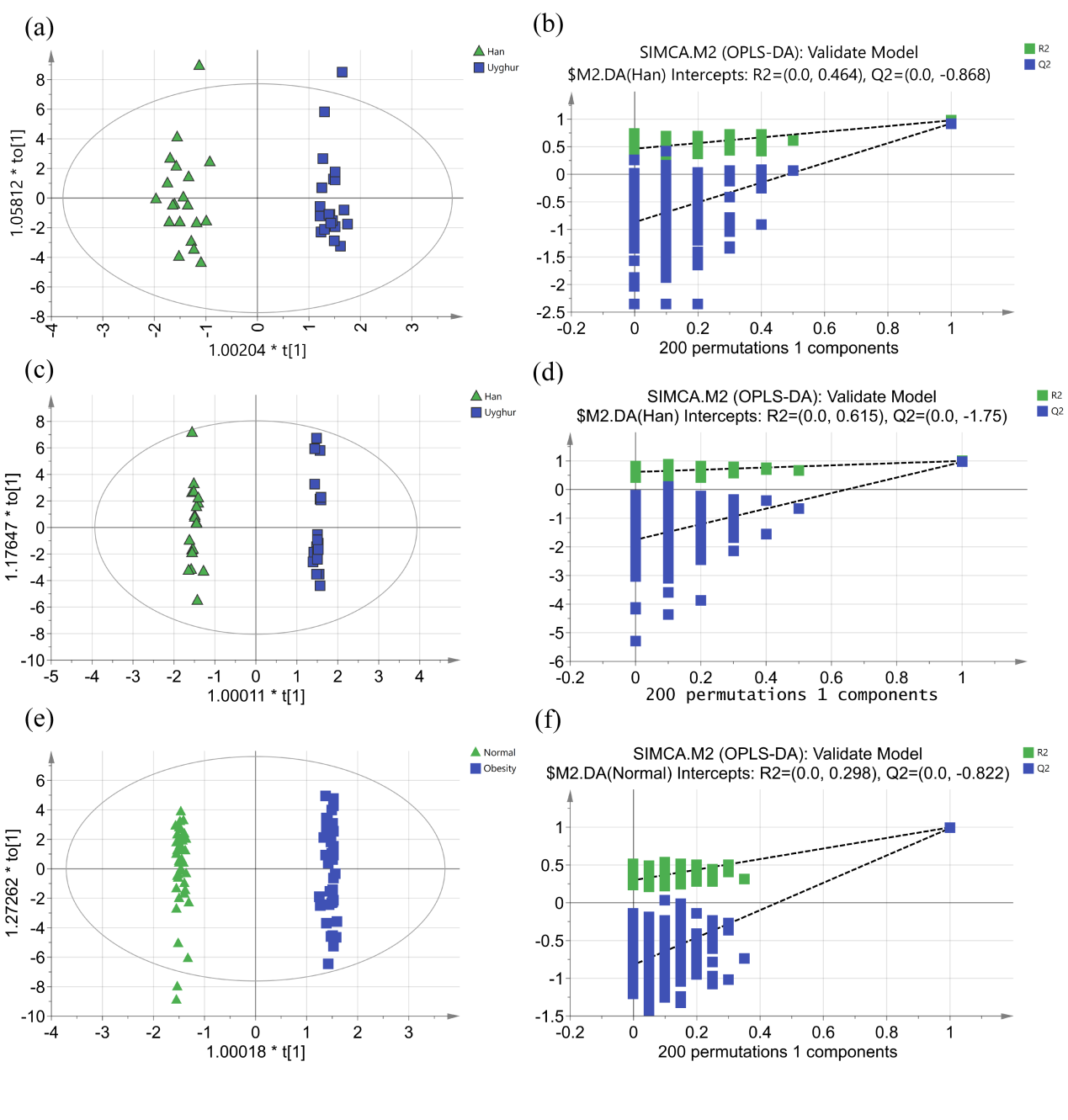


Fig. S1 Orthogonal partial least squares discriminant analysis in different groups between two nationalities. (a)The score plot of the OPLS-DA model shows a clear discrimination between the 20 NW subjects of Hans (green triangle) and 20 NW subjects of Uygurs (blue square) , (b)Permutation test with a permutation number of 200 in the NW groups between two nationalities, (c)The score plot of the OPLS-DA model shows a clear discrimination between the 20 OB subjects of Hans and 20 OB subjects of Uygurs, (d)Permutation test with a permutation number of 200 in the OB groups between two nationalities, (e)The score plot of the OPLS-DA model shows a clear discrimination between the 40 NW subjects and 40 OB subjects of two nationalities, (f)Permutation test with a permutation number of 200 in different groups between two nationalities.

**
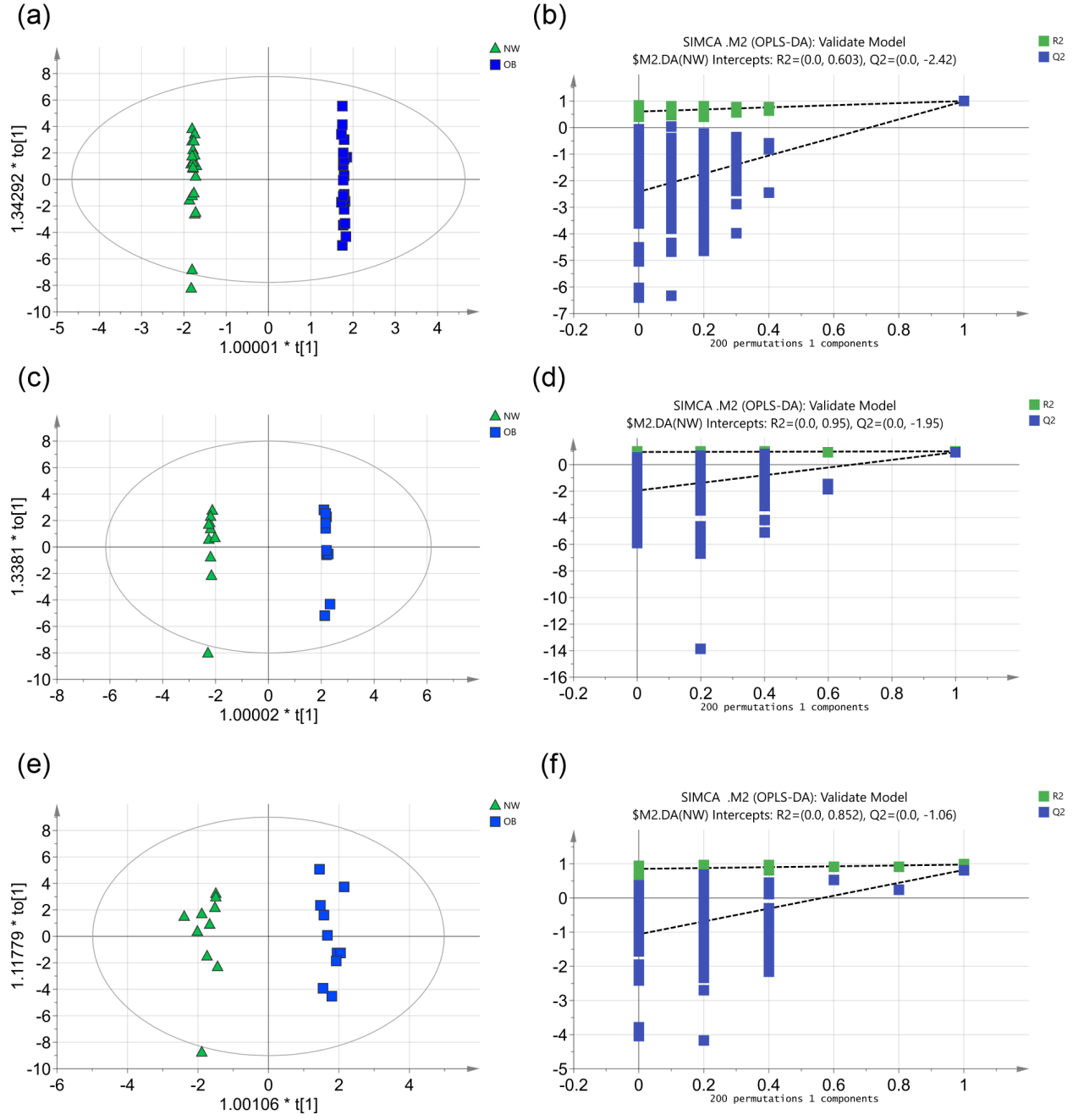
**

**Fig. S2** Orthogonal partial least squares discriminant analysis between NW and OB group in Han nationality. (a)The score plot of the OPLS-DA model shows a clear discrimination between the 20 OB subjects (blue square) and 20 NW subjects (green triangle) in the Han nationality, (b)Permutation test with a permutation number of 200 in Han nationality, (c)The score plot of the OPLS-DA model shows a clear discrimination between the 10 OB subjects and 10 NW subjects in the male group, (d)Permutation test with a permutation number of 200 in the male group, (e)The score plot of the OPLS-DA model shows a clear discrimination between the 10 OB subjects and 10 NW subjects in the female group, (f)Permutation test with a permutation number of 200 in the female group.


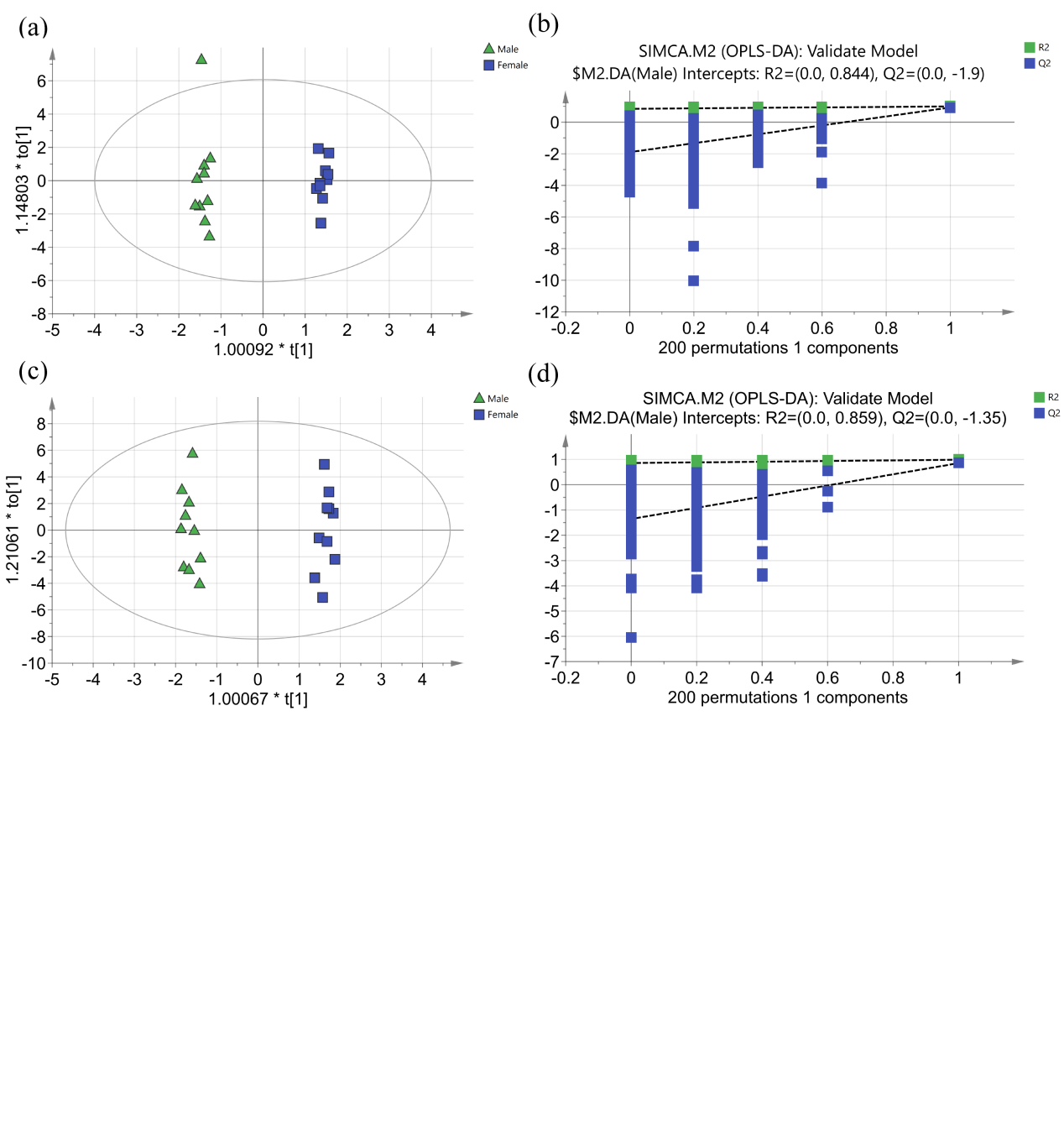


**Fig. S3** Orthogonal partial least squares discriminant analysis between different genders in the Han nationality. (a)The score plot of the OPLS-DA model shows a clear discrimination between the 10 males (green triangle) and 10 females (blue square) in the NW group (b)Permutation test with a permutation number of 200 in the NW group, (c)The score plot of the OPLS-DA model shows a clear discrimination between the 10 males and 10 females in the OB group, (d)Permutation test with a permutation number of 200 in the OB group.


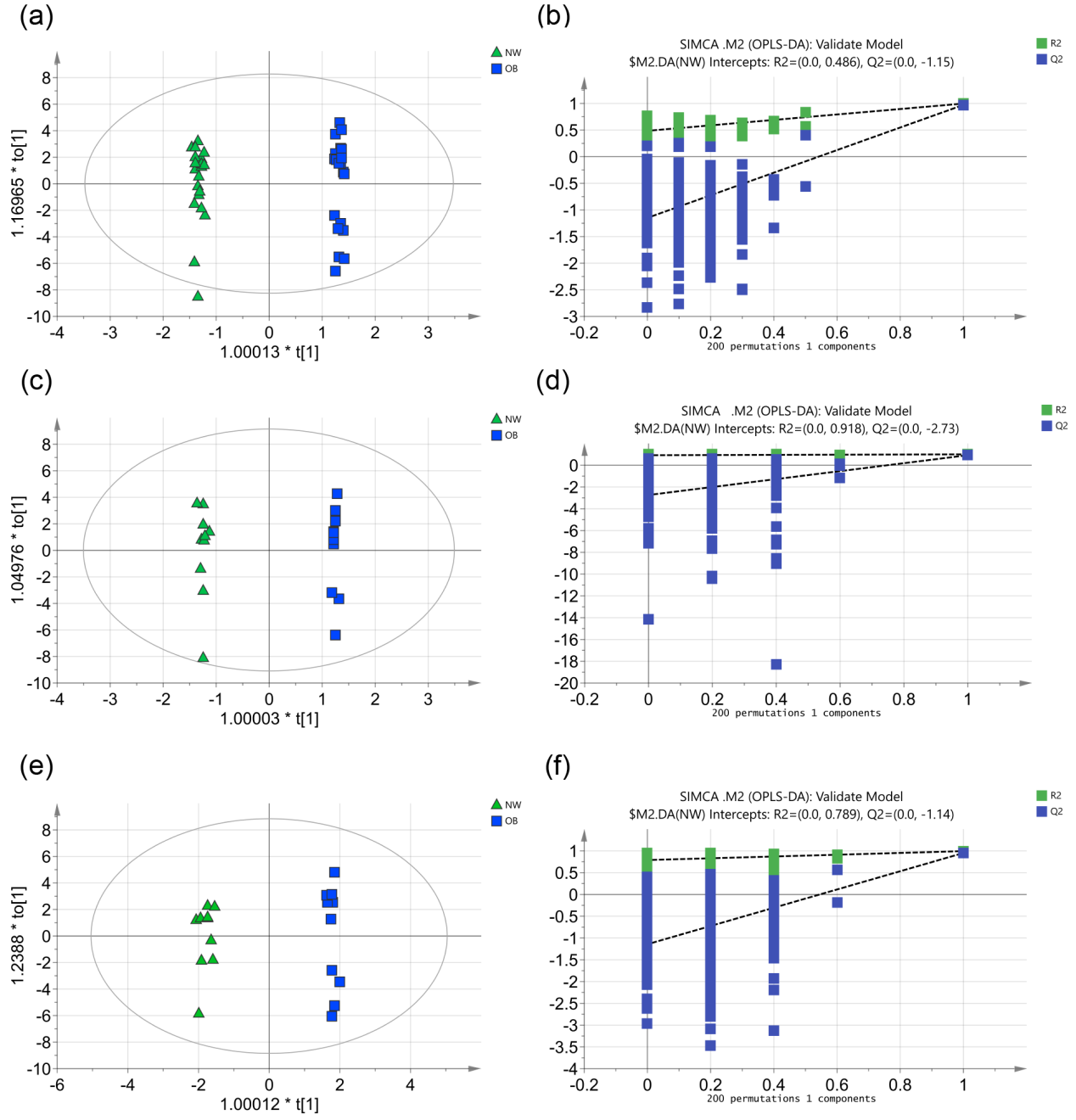


**Fig. S4** Orthogonal partial least squares discriminant analysis between normal NW OB group in Uygur nationality. (a)The score plot of the OPLS-DA model shows a clear discrimination between the 20 OB subjects (green triangle) and 20 NW subjects (blue square) in the Uygur nationality, (b)Permutation test with a permutation number of 200 in the Uygur nationality, (c)The score plot of the OPLS-DA model shows a discrimination between the 10 OB subjects and 10 NW subjects in the males group, (d)Permutation test with a permutation number of 200 in the males group, (e)The score plot of the OPLS-DA model shows a clear discrimination between the 10 OB subjects and 10 NW subjects in the females group, (f)Permutation test with a permutation number of 200 in the females group.


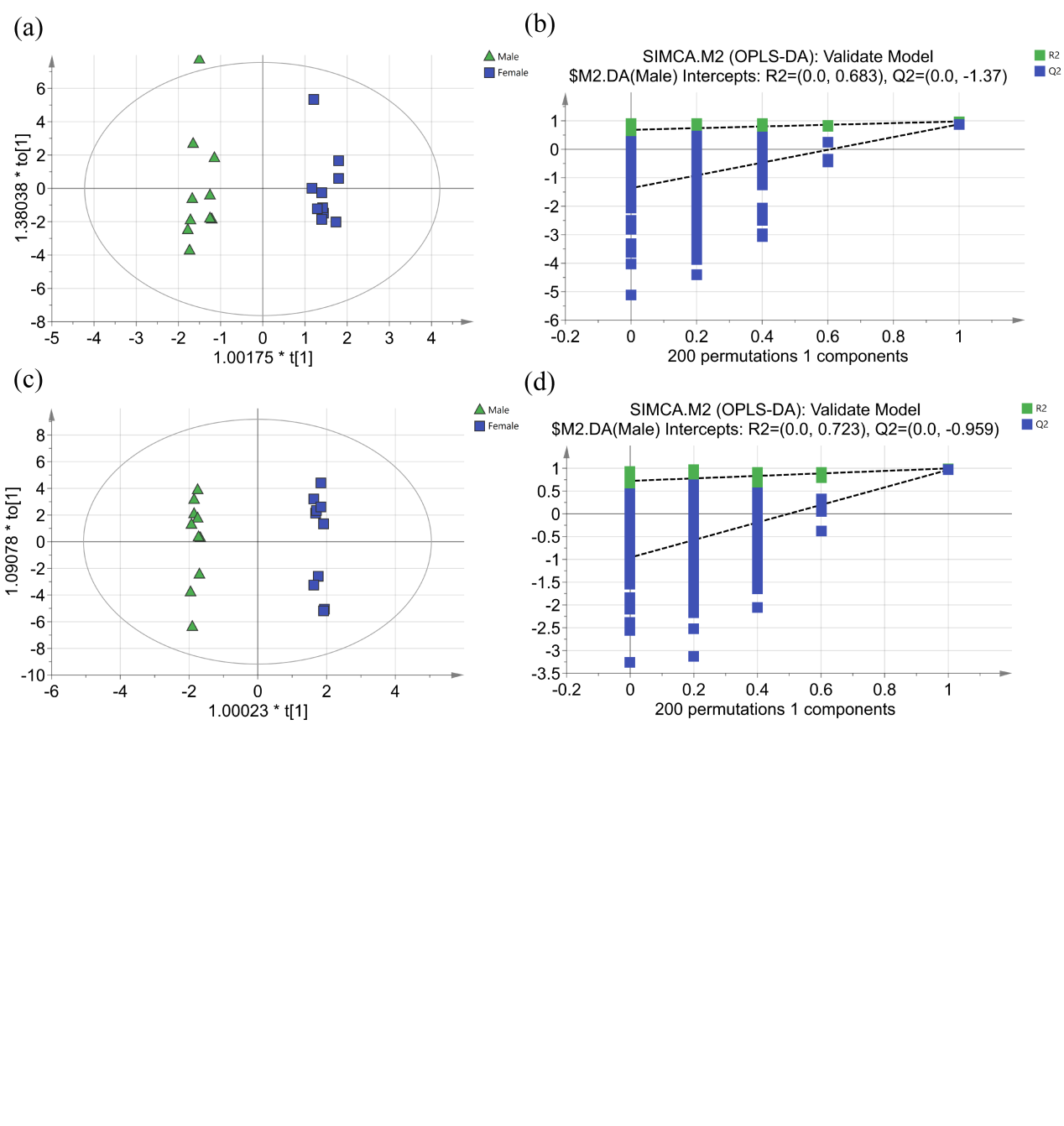


**Fig. S5** Orthogonal partial least squares discriminant analysis between different genders in the Uygur nationality. (a)The score plot of the OPLS-DA model shows a clear discrimination between the 10 males (green triangle) and 10 females (blue square) in the NW group (b)Permutation test with a permutation number of 200 in the NW group, (c)The score plot of the OPLS-DA model shows a clear discrimination between the 10 males and 10 females in the OB group, (d)Permutation test with a permutation number of 200 in the OB group.


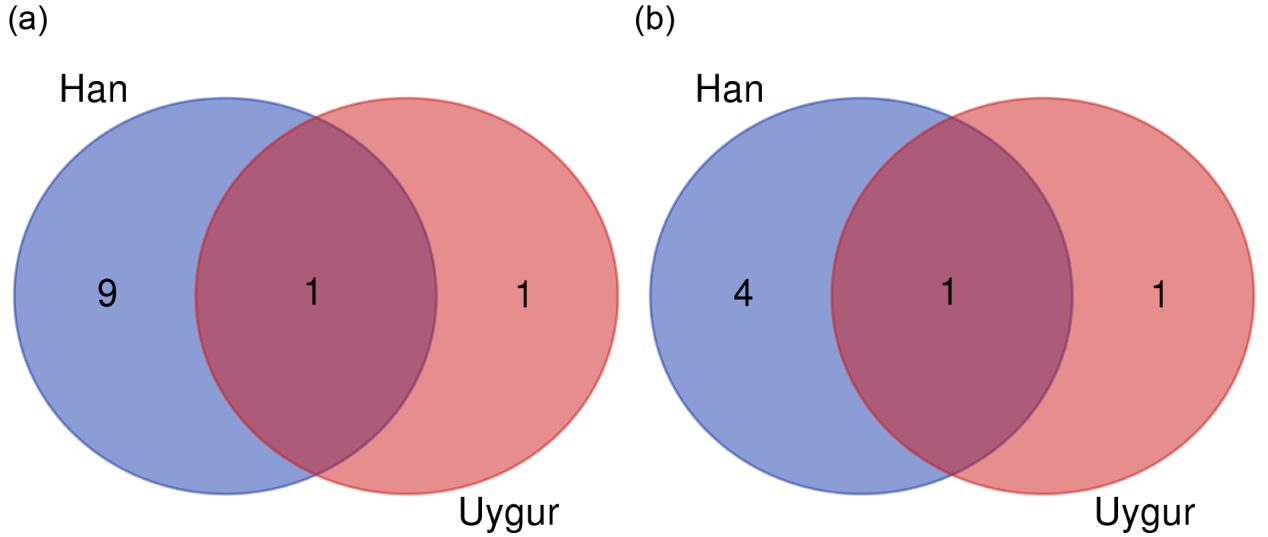


**Fig. S6** The traditional Venn diagram is used to observe the generality and personality differences of FFAs between NW and OB group in two nationalities. (a)On the overall level, observing the generality and personality differences of FFAs between NW and OB group in two nationalities, (b)In the male group, observing the generality and personality differences of FFAs between NW and OB group in two nationalities.

**Table S14.** Diagnostic accuracy of targeted plasma metabolomics in status of obesity.

| **nationality** | **sex** | **classifiers** | **AUROC^*^** | **95%CI** | **RdCV^#^ accuracy** | **2×2^§^**  **Accuracy** | **2×2**  **Sensitivity** | **2×2**  **Specificity** |
| --- | --- | --- | --- | --- | --- | --- | --- | --- |
| **Han** | **Total** | 10-analyte example | 0.781 | 0.597-0.949 | 0.707 | 0.75 | 0.73 | 0.78 |
|  |  | C7:0 | 0.683 | 0.433-0.883 | 0.638 | 0.63 | 0.63 | 0.62 |
|  |  | C8:0 | 0.679 | 0.443-0.893 | 0.599 | 0.73 | 0.71 | 0.74 |
|  |  | C9:0 | 0.694 | 0.454-0.883 | 0.649 | 0.76 | 0.73 | 0.70 |
|  |  | C10:0 | 0.682 | 0.505-0.867 | 0.619 | 0.61 | 0.63 | 0.65 |
|  |  | C11:0 | 0.764 | 0.505-0.939 | 0.731 | 0.78 | 0.75 | 0.73 |
|  |  | C14:0 | 0.685 | 0.479-0.914 | 0.616 | 0.68 | 0.67 | 0.68 |
|  |  | C18:2 | 0.791 | 0.571-0.959 | 0.691 | 0.76 | 0.78 | 0.79 |
|  |  | C20:3 | 0.651 | 0.464-0.847 | 0.536 | 0.68 | 0.68 | 0.67 |
|  |  | C20:4 | 0.737 | 0.535-0.965 | 0.733 | 0.7 | 0.7 | 0.7 |
|  |  | C22:6 | 0.682 | 0.454-0.903 | 0.585 | 0.63 | 0.63 | 0.62 |
|  | **Male** | 10-analyte example | 0.802 | 0.444-1 | 0.727 | 0.75 | 0.73 | 0.78 |
|  |  | C12:0 | 0.608 | 0.222-1 | 0.542 | 0.55 | 0.55 | 0.56 |
|  |  | C14:0 | 0.780 | 0.330-1 | 0.672 | 0.75 | 0.73 | 0.78 |
|  |  | C18:2 | 0.682 | 0.275-1 | 0.628 | 0.65 | 0.64 | 0.67 |
|  |  | C20:3 | 0.765 | 0.444-1 | 0.692 | 0.75 | 0.69 | 0.86 |
|  |  | C20:4 | 0.822 | 0.471-1 | 0.767 | 0.85 | 0.82 | 0.89 |
|  |  | C22:6 | 0.703 | 0.333-1 | 0.665 | 0.7 | 0.75 | 0.67 |
|  | **Female** | 5-analyte example | 0.847 | 0.556-1 | 0.815 | 0.75 | 0.73 | 0.78 |
|  |  | C7:0 | 0.751 | 0.333-1 | 0.672 | 0.7 | 0.7 | 0.7 |
|  |  | C8:0 | 0.654 | 0.333-1 | 0.687 | 0.75 | 0.69 | 0.86 |
|  |  | C9:0 | 0.783 | 0.5-1 | 0.682 | 0.7 | 0.7 | 0.7 |
|  |  | C10:0 | 0.643 | 0.278-1 | 0.608 | 0.65 | 0.64 | 0.67 |
|  |  | C11:0 | 0.904 | 0.693-1 | 0.837 | 0.8 | 0.8 | 0.8 |
| **Uygur** | **Total** | 2-analyte example | 0.581 | 0.362-0.816 | 0.562 | 0.63 | 0.63 | 0.62 |
|  |  | C20:3 | 0.529 | 0.254-0.76 | 0.503 | 0.53 | 0.52 | 0.53 |
|  |  | C20:5 | 0.635 | 0.401-0.878 | 0.608 | 0.6 | 0.63 | 0.58 |
|  | **Female** | 2-analyte example | 0.95 | 0.719-1 | 0.863 | 0.85 | 0.89 | 0.82 |
|  |  | C9:0 | 0.869 | 0.611-1 | 0.793 | 0.85 | 0.89 | 0.82 |
|  |  | C19:0 | 0.858 | 0.5-1 | 0.817 | 0.85 | 0.89 | 0.82 |

n = 20 normal weight males and 20 obesity males in two nationalities, n = 10 normal weight males and 10 obesity males in each nationality, and n = 10 normal weight females and 10 obesity females in each nationality.

* AUROC, area under the receiver operator curve reflects the overall accuracy of diagnosis using these analytes.

# rdCV, repeated random subsample (2/3 in, 1/3 out) double cross-validation.

↵§ Values calculated by standard 2 × 2 contingency table analysis.

**Table S15.** Pathway enrichment analysis of FFAs.

| **Pathway name** | **Total** | **Hits** | ***p*** | **-log(p)** | **Holm p** | **FDR** | **Impact** |
| --- | --- | --- | --- | --- | --- | --- | --- |
| Fatty acid biosynthesis | 49 | 4 | 4.5096E-5 | 10.007 | 0.0036 | 0.0036 | 0.0 |
| Linoleic acid metabolism | 15 | 1 | 0.067 | 2.7092 | 1.0 | 1.0 | 0.656 |
| Arachidonic acid metabolism | 62 | 1 | 0.250 | 1.3864 | 1.0 | 1.0 | 0.217 |

Note: false discovery rates (FDRs)

**Table S16.** Disease enrichment analysis of FFAs.

| **Metabolite Set** | **Total** | **Hits** | **Expect** | ***P* Value** | **Holm p** | **FDR** |
| --- | --- | --- | --- | --- | --- | --- |
| Gestational diabetes mellitus | 3 | 2 | 0.0689 | 0.0014 | 0.389 | 0.243 |
| Hypertension | 12 | 3 | 0.276 | 0.0018 | 0.484 | 0.243 |
| Hyperbaric oxygen exposure | 9 | 1 | 0.207 | 0.19 | 1.0 | 1.0 |
| Cirrhosis | 23 | 1 | 0.528 | 0.421 | 1.0 | 1.0 |

Note: false discovery rates (FDRs)
